# “She finds you abhorrent” - The impact of emotional context information on the cortical processing of neutral faces in depression

**DOI:** 10.3758/s13415-021-00877-x

**Published:** 2021-03-15

**Authors:** Benjamin Iffland, Fabian Klein, Sebastian Schindler, Hanna Kley, Frank Neuner

**Affiliations:** 1 Department of Clinical Psychology and Psychotherapy, Bielefeld University, Postbox 100131, 33501 Bielefeld, Germany; 2 Institute of Medical Psychology and Systems Neuroscience, University of Muenster, Münster, Germany

**Keywords:** Depression, Emotion, Context, Event-related potentials, Childhood maltreatment

## Abstract

Depression is associated with abnormalities in patterns of information processing, particularly in the context of processing of interpersonal information. The present study was designed to investigate the differences in depressive individuals in cortical processing of facial stimuli when neutral faces were presented in a context that involved information about emotional valence as well as self-reference. In 21 depressive patients and 20 healthy controls, event-related potentials (ERPs) were recorded during the presentation of neutral facial expressions, which were accompanied by affective context information that was either self- or other-related. Across conditions, depressive patients showed larger mean P100 amplitudes than healthy controls. Furthermore, mean late positive potential (LPP) amplitudes of depressive patients were larger in response to faces in self-related than in other-related context. In addition, irrespective of self-reference, mean LPP responses of depressive patients to faces presented after socially threatening sentences were larger compared with faces presented after neutral sentences. Results regarding self-reference supported results of previous studies indicating larger mean amplitudes in self-related conditions. Findings suggest a general heightened initial responsiveness to emotional cues and a sustained emotion processing of socially threatening information in depressive patients.

**Supplementary Information:**

The online version contains supplementary material available at 10.3758/s13415-021-00877-x.

## Introduction

Major depressive disorder (MDD) is associated with abnormalities in patterns of information processing and emotional reactivity (Beck et al., 1979; Foti et al., 2010). Negative potentiation theories posit that negative mood states activate specific cognitive structures and increase the processing of negative, schema-congruent stimuli (Scher et al., 2005). This distinctive information processing bias, which may be called “negativity bias,” seems to be most pronounced and detrimental in the context of processing of interpersonal information (Gilboa-Schechtman et al., 2002). The prototype stimulus carrying interpersonal information is the human facial expression. Accordingly, there is considerable evidence that MDD involves specific abnormalities in the cognitive and neural processing of emotional faces (Chen et al., 2014; Gilboa-Schechtman et al., 2002; Gotlib et al., 2004; Leppänen, 2006; Shestyuk et al., 2005; Suslow et al., 2004).

### Context information influences face processing

Because a face is never presented out-of-context, contextual features of a person in a specific situation provide information that may either complement or contradict the actual facial expression (Wieser & Brosch, 2012). Hence, the effective processing of social information requires the integration of both types of information. Studies with nonclinical populations have shown that affective context information, such as affective visual scenes in the background of presented facial expressions, have an impact at early, supposedly automatic and unconscious stages of processing (e.g., N170: Righart & de Gelder, 2006) as well as at later, more conscious processing stages (e.g., Early Posterior Negativity (EPN) or Late Positive Potential (LPP): Diéguez-Risco et al., 2013; Wieser et al., 2014). Additionally, context information is capable of modifying the extent of self-reference of a stimulus or a situation. Self-reference refers to the extent that the information of a stimulus is addressed to the subject. High self-reference may be provided by a descriptive sentence, such as “this person likes you,” whereas a third-person statement, such as “this person likes someone” provides no self-reference. Regardless of the valence of the stimulus and the context information, the LPP (Wieser et al., 2014) as well as the EPN (Klein et al., 2015) were modulated by the extent of self-reference of the context. Moreover, an interaction between self-reference and valence, with self-relevant neutral context information leading to most pronounced LPP amplitudes was found in nonclinical individuals (Klein et al., 2015). In line with previous research (Fields & Kuperberg, 2012; Hirsh & Inzlicht, 2008), this finding has been interpreted as being indicative of a higher motivation to evaluate the valence of neutral faces because of their ambiguous manner.

### Emotion and face processing in depressive individuals

Neuroimaging studies found abnormalities in areas associated with self-referential processing in depressive patients (e.g., default mode network structures or rostral anterior cingulate cortex (Sheline et al., 2009; Wagner et al., 2015). The authors suggested that depressed patients are less capable of shifting their attention away from negative self-related stimuli. However, electroencephalographic (EEG) studies that examine potential abnormalities in context integration in the processing of facial stimuli in depressed patients are still scarce. Therefore, we planned to extend previous findings regarding cortical processing of facial stimuli presented in a context containing information about the affect as well as self-reference to a sample of depressed individuals (Klein et al., 2015; Wieser et al., 2014).

With respect to previous studies, the present study focused on N170, EPN, and LPP components (Klein et al., 2015; Righart & de Gelder, 2006; Wieser et al., 2014). The N170 is a prominent component that is reliably triggered by face stimuli. This process seems to be altered by depression (Dai & Feng, 2012), albeit some studies could not find any differences at this stage (Foti et al., 2010; Jaworska et al., 2012). However, activation of the N170 in depressive patients seems to be dependent on the affective expression of faces that patients are confronted with. In a study by Chen et al. (2014), subjects with major depression showed a decreased N170 for the processing of neutral and happy faces, but an increased N170 for sad faces. The authors argued that these results may show the altered emotional processing and the specific cognitive bias for negative emotions.

The LPP has been associated with emotional processing of faces and other stimuli in a motivated attention framework (Frenkel & Bar-Haim, 2011; Hajcak et al., 2009; Schupp et al., 2004), particularly with the decoding and appraisal of affective meaning (Schupp et al., 2006; Wessing et al., 2013). Compared with healthy controls, LPP responses to unpleasant emotional stimuli have been shown to be reduced in depressed individuals (Foti et al., 2010; Kayser et al., 2000; MacNamara et al., 2016). Within depressive individuals, however, responses to emotional faces and to self-referential emotional words involved enhanced LPPs (Auerbach et al., 2015; Burkhouse et al., 2017). Particularly, in a self-referential encoding paradigm, depressed subjects showed greater early LPP positivity to negative versus positive words (Shestyuk & Deldin, 2010). Similarly, Jaworska et al. (2012) reported augmented LPPs in reaction to sad faces in depressive subjects suggesting an attentional bias towards mood-congruent stimuli.

The EPN is a mid-latency component usually starting at around 200 ms post-stimulus that has been connected to enhanced emotional processing (Hajcak et al., 2010; Wieser et al., 2014). To date, only poor evidence links depression to EPN activity. Consistent with the negative potentiation hypothesis, though, depressed individuals have recently been reported to exhibit enhanced EPN amplitudes in reaction to both negative, neutral, and positive stimuli compared with nondepressed subjects (Connell et al., 2015). The authors proposed that depression was associated with greater early attentional engagement for emotionally valenced stimuli.

In addition to the N170, the P100, a positive deflection occurring at occipital sites at around 100 ms after stimulus onset, has been shown to be associated with face processing (Herrmann et al., 2005; Righart & de Gelder, 2008). While the N170 is reported to reflect a stage of configural processing, the P100 was suggested to reflect a stage of face detection (Itier & Taylor, 2004). Contrasting with the N170, however, information on modulations of the P100 by depression in the processing of facial stimuli has been scarce. With respect to findings indicating that depressed individuals show greater P100 amplitudes (Auerbach et al., 2015) and decreased P100 latency (Yang et al., 2011) in response to negative self-referential words, it was suggested that the P100 component reflects a promising means of assessing self-referential biases in depression (Auerbach et al., 2015). Therefore, as a novelty, analyses of the P100 component in the processing of facial stimuli were included in the present study.

### Emotion processing in depression related to childhood maltreatment

Recently, different clinical subtypes of depression have been proposed (Sharpley & Bitsika, 2014). Most prominently, a subtype of depression that is related to experiences of childhood maltreatment was suggested which is characterized by an earlier onset, a more chronic course, and more suicide attempts (Andersen & Teicher, 2008; Zisook et al., 2007). However, research examining its underlying physiological characteristics is still at its beginnings. Therefore, for exploratory purposes, the present study sought to examine the influence of childhood maltreatment on information processing in depressive patients. In doing so, we planned to investigate the influence of various types of physical, emotional, and sexual childhood maltreatment as well as relational peer victimization differentially. Accordingly, differential effects of various forms of childhood maltreatment on psychopathology, emotional functioning as well as facial emotion processing were previously reported (Dong et al., 2004; Humphreys & Zeanah, 2015; Iffland & Neuner, 2020; McLaughlin et al., 2014; Roth et al., 2018; Teicher & Samson, 2013; Zeanah & Sonuga-Barke, 2016). Addressing previous studies reporting enhanced ERPs in maltreated children at P100 and P3b components (Pollak et al., 2001; Pollak & Tolley-Schell, 2003; Shackman et al., 2007), the present study sought to particularly examine associations of the P100 and P3b components and various types of childhood maltreatment in patients with depression.

### Study objective and hypotheses

As an extension of prior work in healthy individuals (Klein et al., 2015), we examined whether depressive individuals show abnormal cortical processing of stimuli presented in a context containing information about the affect as well as self-reference. In healthy participants, we expected to find similar modulations of the N170, EPN, and LPP as presented by Klein et al. (2015). Also, we predicted a general enhancement in a) socially threatening context information in comparison with physically threatening and neutral context information, and in b) self-relevant stimuli compared to other-relevant stimuli. In depressive individuals, we assumed that depressive individuals present with a lack of adaptive context integration, which may lead to a generally heightened impact of negative, self-related context information (Wagner et al., 2015). More specifically, based on previous findings (Chen et al., 2014; Wagner et al., 2015), we hypothesized an enhanced activity on the N170 in depressed individuals in response to faces presented in a negative, self-related context, while healthy controls show more pronounced N170 amplitudes to neutral and other-related context information (Dai & Feng, 2012). With respect to the LPP, we expected that depressive subjects exhibit weaker responses to faces than healthy controls (Foti et al., 2010; Kayser et al., 2000; MacNamara et al., 2016). Within the group of depressive individuals, we assumed most pronounced LPP responses in reaction to faces with negative, self-related context information (Auerbach et al., 2015; Burkhouse et al., 2017; Jaworska et al., 2012; Shestyuk & Deldin, 2010). Based on previous research regarding the EPN component (Connell et al., 2015), we expected larger EPN responses in depressed individuals, irrespective of context information. Finally, we hypothesized that P100 amplitudes were largest in reaction to negative, self-related stimuli in the depressive sample, while healthy individuals’ P100 responses were not different in response to different emotional contexts (Auerbach et al., 2015). For exploratory purposes, associations of the P100 and P3b components and childhood maltreatment in patients with depression were examined.

## Methods

### Participants

Participants were recruited through the Outpatient Psychotherapy Clinic of Bielefeld University and through newspapers and magazines as well as bulletins at the campus of Bielefeld University. Depressive patients recruited from the Outpatient Clinic were currently on the waiting list for therapy and had shown signs of depression in an initial clinical interview. In order to validate a current diagnosis of depression, depressed patients underwent an extensive structured clinical interview (German version of the SCID-I: Wittchen et al., 1997), administered by trained clinical psychologists. Exclusion criteria for depressive patients included (a) any current DSM-IV Axis I psychiatric disorder other than major depression without a co-morbid depressive disorder, (b) evidence of a current substance abuse or dependence, (c) evidence of current or past psychosis, and (d) evidence of acute suicidal intentions or ideation. Participants recruited from newspapers and bulletin boards were used as a healthy control group and underwent a brief structured clinical interview (German version of the Mini-International Neuropsychiatric Interview (M.I.N.I.); Sheehan et al., 1998) before the experiment to make sure that they had no current or known lifetime history of axis I DSM-IV psychiatric disorders (American Psychiatric Association, 2000). Depressive patients (*n* = 21) had a mean age of 39.48 years (standard deviation [SD] = 14.43, range 18–58 years, 9 females), whereas participants of the healthy control group (*n* = 20) had an average age of 39.20 years (SD = 16.02, range 20–66, 8 females). Of the 21 patients with depression, 14 were diagnosed with a recurrent MDD and 7 were diagnosed with a first episode MDD. Comorbid DSM-IV diagnoses in the group of depressed patients, based on the SCID-I-assessment, were specific phobia (n = 3), social phobia (n = 3), generalized anxiety disorder (n = 1), posttraumatic stress disorder (n = 2), panic disorder without agoraphobia (n = 1), hypochondriasis (n = 1), and anorexia (n = 1). Current use of psychotropic medication was reported by 47.6% (*n* = 10) of the depressive patients (antidepressant: *n* = 10; anxiolytic: *n* = 3). Participants of the healthy control group received either course credits (when studying at Bielefeld University) or financial compensation for participation. In the depressive sample, participants did not receive financial compensation. Instead, participants were offered prompt and detailed psychological diagnostic sessions within the present study. Afterwards, depressive patients received detailed written and oral diagnostic feedback. Participants in both groups had normal or corrected-to-normal vision. All subjects received and signed an informed consent before the experiment. The experimental procedure was approved by the Ethics Committee of Bielefeld University. The study was in accordance with the declaration of Helsinki. Table 1 presents participants’ means on the assessments.

**Table 1:** Participants’ characteristics and mean values on the assessments (*N* = 41)

	Depressive patients (*n* = 21)	Healthy controls (*n* = 20)	*p*
Age, *M* (SD, range)	39.48 (14.43, 18-58)	39.20 (16.02, 20-66)	0.954
Gender, % female (*n*)	42.9 (9)	40.0 (8)	0.853^a^
Family status, *% single* (*n*)	19.0 (4)	60.0 (12)	0.007^a^*
Years of education, *M* (SD)	14.57 (4.06)	16.35 (4.84)	0.209
Educational level, % high school graduation and higher (*n*)	66.7 (14)	85.0 (17)	0.172^a^
Beck Depression Inventory, *M* (SD)	25.33 (8.93)	3.75 (2.53)	<0.001*
Brief Symptom Inventory^b^, *M* (SD)	1.21 (.53)	.26 (.19)	<0.001*
Childhood trauma questionnaire, *M* (SD)	42.10 (18.33)	34.75 (9.72)	0.117
Physical abuse, *M* (SD)	6.71 (4.23)	5.85 (1.57)	0.396
Emotional abuse, *M* (SD)	9.24 (6.39)	7.75 (3.43)	0.357
Physical neglect, *M* (SD)	8.19 (3.87)	6.70 (2.27)	0.143
Emotional neglect, *M* (SD)	12.52 (5.47)	8.70 (3.13)	0.009*
Sexual abuse, *M* (SD)	5.43 (1.43)	5.75 (1.74)	0.522
Relational peer victimization^c^	9.90 (8.83)	6.80 (5.68)	0.187

^a^Chi-square-test. ^b^Global Severity Index (GSI). ^c^*Fragebogen zu belastenden Sozialerfahrungen* [Adverse Social Experiences Questionnaire] (FBS; Sansen et al., 2013); *p < 0.05.

### Instruments

#### Symptoms of depression

Symptoms of depression were measured using the German version of the Beck Depression Inventory (BDI-II; Hautzinger et al., 2006). The self-report measure consists of 21 items. The items are rated on a 4-point scale indicating the severity of symptoms. Higher scores indicate more severe depressive symptoms. Standardized cutoff scores indicate sumscores >13 as mild depression. The BDI-II has shown good psychometric properties in nonclinical and clinical samples (Kühner et al., 2007). In the current sample, internal consistency of the BDI-II was excellent (Cronbach’s *α* = 0.95).

#### General psychopathology

As a measurement of psychopathology and psychological distress in general, we used the German version of the Brief Symptom Inventory (BSI; Franke, 2000). The BSI is a short form of the Symptom Checklist 90 (SCL-90) and consists of 53 items, producing the same nine primary symptom dimensions (somatization, obsessive-compulsive symptoms, interpersonal sensitivity, depression, anxiety, hostility, phobic anxiety, paranoid ideation, and psychoticism). Additionally, three global indices measure general psychological distress: the Global Severity Index (GSI); the Positive Symptom Total (PST); and the Positive Symptom Distress Index (PSDI). The items are rated on a 5-point Likert scale, ranging from 0 (not at all) to 4 (extremely) and relates to the experience of the past 7 days, including the day of assessment. In the present study, the GSI was used to indicate participants’ psychological distress. The BSI showed high internal consistency in the current sample (Cronbach’s *α* = 0.97).

#### Childhood maltreatment

The German version of the Childhood Trauma Questionnaire (CTQ; Wingenfeld et al., 2010) was used to assess different types of childhood maltreatment (sexual abuse, emotional neglect, emotional abuse, physical neglect, and physical abuse). The items are rated from 1 (never true) to 5 (very often true) with a possible range of subscale scores from 5 to 25. The psychometric properties of the German version are similar to the original version, and it has been shown to be a reliable and valid screen for childhood maltreatment. In the current sample, internal consistency was excellent for all items (Cronbach’s α = 0.94). Similarly, internal consistency of the subscales sexual abuse, emotional neglect, emotional abuse, and physical abuse was good to excellent (all Cronbach’s α’s > 0.83). The subscale physical neglect, however, showed only a questionable internal consistency (Cronbach’s α = 0.69), which is in line with prior reports of weak psychometric properties of this subscale of the German version (Klinitzke et al., 2012).

#### Relational peer victimization

Relational peer victimization was assessed using the *Fragebogen zu belastenden Sozialerfahrungen* [Adverse Social Experiences Questionnaire] (FBS; Sansen et al., 2013). The FBS consists of 22 items describing aversive social situations, such as rejection, exclusion, being laughed at, insulted, and teased by peers. For each situation, respondents were asked whether or not they have experienced this situation during childhood (age 6-12 years) or adolescence (age 13-18 years). The total score is calculated as a sum of “Yes” responses across both age periods and ranges from 0 to 44. The total score of the FBS presented with a satisfying stability over a 20-month period (r = 0.89) (Sansen et al., 2013). In the current sample, internal consistency of the FBS was excellent (Cronbach’s α = 0.91).

### Stimulus Material

Photographs of faces of 22 different Caucasian individuals (11 females, 11 males) were taken from the Radboud Faces Database (Langner et al., 2010). The RFDB offers a variety of different facial expressions, representing different emotions, as well as neutral facial expressions—all of them presented at different camera angles. For the present study, only neutral faces with frontal orientation were included. The pictures were resized to a resolution of 371 × 556 pixels. There was no further standardization in terms of contrast or luminance, because the randomized presentation of all faces would rule out all possible systematic influences.

The context stimuli consisted of 18 German sentences from three different valence categories (physically threatening, socially threatening, and neutral) and were couched in a self-related and other-related fashion as well (e.g., “*He wants to hit you.”* vs. “*He wants to hit someone.”*). Physically threatening sentences described situations where an aggressor intends to conduct actual physical violence or uttering violent threats (e.g., *“He wants to smash your face in*.*”*). Socially threatening sentences were focused on intimidation or the impending loss of social belonging or rank (e.g., “*She finds you abhorrent.”*). Neutral sentences were characterized by descriptions of nonjudgmental or nonthreatening behaviors or situations (e.g., “*He is sitting next to you.”*). The stimulus set has been developed by Klein et al. and has been evaluated regarding differences in arousal and valence (Klein et al., 2015).

### Procedure

To test our assumptions, we presented three different classes of emotions (physically threatening, socially threatening, and neutral) as written statements that preceded faces with inherently neutral expressions. As a second factor, the context sentences were either directed to the participant or to other persons to modulate the self-referential aspect of the context sentences. Each context sentence and face stimulus was randomly paired for each trial to be able to examine a short-term, one-time influence instead of learning effects.

For the presentation of the experiment, the software package Inquisit 4.0.3 (Millisecond Software, Seattle, WA) was used. The experiment was shown on a 19-inch-TFT-monitor (60-Hz refresh rate), which was located approximately 60 cm in front of the participant. The participants were asked to focus their attention on the center of the screen and passively view the displayed pairs of faces and sentences. The paradigm has been established in a previous study (Klein et al., 2015). Each sentence (self-related/physically threatening, self-related/socially threatening, self-related/neutral, other-related/physically threatening, other-related/socially threatening, other-related/neutral) was pseudo-randomly paired with one of the 22 faces. Each sentence was automatically worded in an appropriate gender-specific fashion (e.g., female face – female personal pronoun). Because the experimental setting itself did not provoke any distractions that would divert focus from the screen, we did not include a fixation cross in our design. In order to ensure that differences in the ERPs are only caused by the influence of the sentences, there was no fixed combination of any face-sentence pair. The experiment consisted of six blocks, each block contained 48 randomly chosen trials, leading to a total number of 288 trials. Each of the six conditions (self-related/physically threatening, self-related/socially threatening, self-related/neutral, other-related/physically threatening, other-related/socially threatening, other-related/neutral) was presented 48 times. Each trial started with the presentation of a sentence for 2,900 ms (interstimulus-interval randomized between 900-1,500 ms), followed by a face, which was presented for 500 ms. The intertrial-interval consisted of an empty gray background and varied randomly between 1,900 and 2,600 ms. Between each block, there was a short break for approximately 2 minutes.

### EEG preprocessing and statistical analyses

EEG data was continuously recorded from 128 active electrodes (BioSemi Active Two System; www.biosemi.com), online referenced to the Common Mode Sense (CMS) - Driven Right Leg (DRL) ground. Four additional electrodes were placed above and below the right eye as well as on the outer canthi of both eyes to record horizontal and vertical eye-movements. Online data were recorded with 2,048 Hz.

Preprocessing was performed using SPM8 for EEG data (http://www.fil.ion.ucl.ac.uk/spm). Offline data was re-referenced to the average reference, downsampled to 250 Hz and bandpass-filtered between 0.166 and 30 Hz with a fifth order zero-phase. Filtered data were epoched from 100 ms before to 600 ms after presentation of the neutral faces and baseline corrected (100 ms to 0 ms prestimulus). In a first step, artifact trials exceeding a high threshold (>150 μV) were rejected. Finally, data were averaged using the robust averaging algorithm (Litvak et al., 2011). This averaging method is a unique feature of SPM, down weighting outliers for each channel and time point, which preserves a higher number of trials while controlling for a variety of possible artifacts. We used the recommended weighting function, which preserves approximately 95% of the data points drawn from a random Gaussian distribution. After averaging, visual inspection of the data was performed and bad channels were identified. On average 1.3% of all sensors were interpolated (*M =* 1.68; SD = 2.02). On average, 4.33 trials were rejected keeping 43 trials for each single condition. A mixed repeated measure ANOVA showed there were no differences in the number of rejected trials between controls and patients (*F*(1, 39) = 2.20, *p* = 0.146, η^2^ = 0.053), nor between self- and other-related context (*F*(1, 39) < 0.01, *p* = 0.957, η^2^ < 0.001), nor between the three emotional valences (*F*(2, 78) = 2.19, *p* = 0.119, η^2^ = 0.053). Similarly, no significant interactions between self-reference and emotion (*F*(2, 78) = 0.76, *p* = 0.471, η^2^ = 0.019), group and self-reference (*F*(1, 39) = 0.39, *p* = 0.537, η^2^ = 0.010), group and emotion (*F*(2, 78) = 2.65, *p* = 0.077, η^2^ = 0.064), and between group, self-reference, and emotion (*F*(2, 78) = 0.73, *p* = 0.485, η^2^ = 0.018) were found.

Components and electrode clusters based on previous studies of contextual influences on face perception (Klein et al., 2015; Wieser et al., 2014). To validate expected time windows in the current sample, we used a collapsed localizer for the P1 and N170 (Luck & Gaspelin, 2017) and collapsed negative-neutral difference waves for the EPN and LPP. However, because difference waves for the EPN did not show a profound negativity, the EPN component was captured around the N2 wave amplitude using the same electrode set as for the N170 (Bruchmann et al., 2020; Schindler et al., 2019, 2020). We investigated differences on early (P100, N170), mid-latency (EPN), and late stages of face processing (LPP). The P100 was measured between 90 and 120 ms, using a parieto-occipital cluster (12 electrodes: PPOz, POz, POOz, Oz, P1, PPO3, PO3, POO3, O1, P2, POO4, PO4, O2). The N170 was detected between 120 and 170 ms and the EPN between 250 and 450 ms after stimulus onset. For both components, two bilateral symmetric occipital sensor clusters of eight electrodes each were examined (left: I1, OI1, O1, PO9, PO9h, PO7, P9, P7; right: I2, OI2, O2, PO10, PO10h, PO8, P10, P8). Finally, LPP effects were investigated between 450 and 600 ms over centroparietal locations (20 electrodes: CCP5, CCP3h, CCP3, CCP4, CCP4h, CCP6, CPz, CPP3, CPPz, CPP4, P5, P3, P1, Pz, P2, P4, P6, PO3, POz, PO4). For the exploratory analyses, the P3b was detected between 300 and 450 ms. The P3b showed a maximum over centroparietal electrodes (13 electrodes: FCC1, FCC2, Cz, CCP1, CCPz, CCP2, CP1, CPz, CP2, CPPz, P1, Pz, P2). In all statistical analyses, mean voltage activity in the given time intervals for the mentioned electrodes was used.

To ensure that ERN score averages were reliable, the number of trials needed to achieve a reliability threshold of 0.80 was calculated for each diagnostic group (Table 2). A reliability threshold of 0.80 was deemed acceptable based on established guidelines for ERP score reliability (Clayson & Miller, 2017a). ERP score reliability as a function of the number of trials needed for a stable average and diagnostic group was examined using the ERP Reliability Analysis (ERA) Toolbox v 0.3.2 (Clayson & Miller, 2017b). The ERA Toolbox calculated ERN score dependability based on algorithms from generalizability theory and used CmdStan v 2.10.0 (Stan Development Team, 2016) to implement the analyses in Stan (Carpenter et al., 2017). Furthermore, Spearman-Brown split-half reliabilities were calculated for all conditions on all components (Table 2). All components showed acceptable to excellent reliabilities. However, because reliability of the LPP component was badly affected, data of one participant of the depressive group was excluded from LPP analyses.

**Table 2:** ERP reliability scores for each ERP component

ERP component	trial numbers^a^	estimated dependability [CI]	ICC^b^ [CI]	total	Self-related socially threatening	Self-related physically threatening	Self-related neutral	Other-related socially threatening	Other-related physically threatening	Other-related neutral
				ρ^c^	ρ^c^	ρ^c^	ρ^c^	ρ^c^	ρ^c^	ρ^c^
P1 HC	33	0.97 [0.95, 0.98]	0.12 [0.07, 0.21]	0.97	0.73	0.88	0.95	0.86	0.73	0.89
P1 MDD	15	0.99 [0.98, 0.99]	0.23 [0.14, 0.37]	0.98	0.96	0.94	0.94	0.92	0.90	0.94
N170 HC	10	0.99 [0.98, 1.00]	0.32 [0.20, 0.47]	0.99	0.92	0.94	0.94	0.94	0.95	0.97
N170 MDD	7	0.99 [0.99, 1.00]	0.41 [0.27, 0.57]	0.99	0.97	0.96	0.95	0.95	0.94	0.97
EPN HC	12	0.99 [0.98, 0.99]	0.27 [0.16, 0.44]	0.99	0.88	0.91	0.91	0.91	0.89	0.95
EPN MDD	15	0.99 [0.98, 0.99]	0.23 [0.14, 0.38]	0.98	0.95	0.90	0.93	0.94	0.79	0.94
P3b HC	12	0.99 [0.98, 0.99]	0.26 [0.15, 0.43]	0.98	0.86	0.96	0.94	0.92	0.91	0.94
P3b MDD	23	0.98 [0.96, 0.99]	0.16 [0.09, 0.28]	0.96	0.87	0.89	0.58	0.90	0.88	0.94
LPP HC	18	0.98 [0.97, 0.99]	0.20 [0.11, 0.33]	0.97	0.86	0.90	0.86	0.81	0.84	0.92
LPP MDD	35	0.97 [0.94, 0.99]	0.11 [0.06, 0.20]	0.97	0.84	0.77	0.84	0.86	0.88	0.78

^a^Trials needed to achieve a dependability point estimate of 0.80. On all components, all participants of both groups survived the trial cutoff (100%). ^b^ Intraclass correlation coefficients (ICC). This measure captures both between-person as well as within-person variance. ^c^ Spearman-Brown split-half reliabilitiy. Larger numbers indicate more reliable ERP components.

Statistical tests on EEG data and data visualization were performed with EMEGS (http://www.emegs.org/, Peyk et al., 2011). For statistical analyses, 2 (group: depressive patients vs. healthy controls) x 2 (self-reference: self-related vs. other-related) x 3 (emotional valence: socially threatening, neutral, physically threatening sentences) repeated measures ANOVAs were conducted to investigate differences between conditions in all time windows of interest. Because stronger effects over right sensors are found in a number of studies on emotion processing or contextual threat in the N170 and EPN time window (Hinojosa et al., 2015; Wieser et al., 2010), laterality was included as an additional factor (laterality: left vs. right). Effect sizes were calculated for all statistical tests (Cohen, 1988). When necessary, additional post-hoc ANOVAs as well as paired *t*-tests were conducted separately for different emotional valences, differently referenced conditions, and depressive patients versus healthy controls. Post-hoc *t*-tests were adjusted for multiple comparisons using False discovery rate (FDR) correction (Benjamini & Hochberg, 1995). When Mauchly’s test indicated violation of the sphericity assumption, Greenhouse–Geisser corrections were applied and original degrees of freedom together with Greenhouse–Geisser ε are reported. Additionally, when bivariate Spearman rank correlations indicated significant associations of mean ERP amplitudes and measures of childhood maltreatment, all ANOVAs were performed as analyses of covariance (ANCOVAs) with childhood maltreatment scores serving as covariates. As the pattern of results did not change, only ANOVAs are reported. For exploratory purposes, correlation analyses were conducted to examine the associations of childhood maltreatment, measured by scores on the CTQ and FBS, with mean ERP amplitudes of the P100 and P3b components in depressive patients. To control for severity of depressive symptoms and because all maltreatment scales were not normally distributed (all *W*’s < 0.89, all *p*’s < 0.05), partial Spearman’s rank order correlations were calculated with the BDI score serving as control variable. The results are reported as rho coefficients with their respective *p* values. Statistical significance was set at *p* < 0.05.

## Results

### Preliminary analyses

As expected, depressive patients showed significantly higher scores on psychopathology as measured by the BDI and BSI (Table 1). Additionally, depressive patients reported higher levels of emotional neglect, while reports of other forms of childhood maltreatment as well as relational peer victimization did not differ significantly between the depressive and the control group. Moreover, groups differed with respect to their family status, with healthy controls reporting more frequently being single. Furthermore, preliminary analyses showed significant bivariate associations between measures of psychopathology and childhood maltreatment and ERP amplitudes across conditions, particularly on the P100 component. See Supplement 1 for more details on bivariate Spearman rank correlations between measures of childhood maltreatment and psychopathology and ERP mean amplitudes.

In addition, in a preliminary analyses step ERP amplitudes of the healthy control group were analyzed to examine whether findings of Klein et al. (2015) could be replicated in the present sample (Supplement 2). Here, findings on the N170 component fully replicated the findings by Klein et al. (2015), whereas results regarding EPN and centroparietal LPP slightly differed. As presented by Klein et al. (2015), a main effect of self-reference was found on the EPN. However, laterality did not have a significant effect on EPN amplitudes in the present sample. In line with findings regarding the centroparietal LPP indicated by Klein et al. (2015), the interaction between self-reference and valence also was significant in the present analysis.

### Event-related brain potentials

Means and standard deviations of the mean amplitudes of the event-related brain potentials (ERPs) components are presented in Table 3.

**Table 3:** Means and standard deviations of mean amplitudes of ERP components

	Depressive patients (*n* = 21)	Healthy controls (*n* = 20)
	*M* (SD)	*M* (SD)
*P100 (90-120 ms)*		
Self-related socially threatening	4.32 (2.64)	2.81 (2.08)
Self-related physically threatening	4.28 (2.64)	2.68 (2.05)
Self-related neutral	4.27 (2.68)	2.45 (2.16)
Other-related socially threatening	4.10 (2.52)	2.54 (2.15)
Other-related physically threatening	4.31 (2.36)	2.54 (2.01)
Other-related neutral	3.94 (2.63)	2.64 (2.15)
*N170 (120-170 ms)*		
Self-related socially threatening, left	−3.29 (4.16)	−3.11 (3.09)
Self-related socially threatening, right	−3.79 (5.01)	−4.97 (4.29)
Self-related physically threatening, left	−3.34 (3.95)	−2.90 (3.15)
Self-related physically threatening, right	−4.08 (4.60)	−4.87 (4.60)
Self-related neutral, left	−3.24 (4.40)	−3.37 (3.18)
Self-related neutral, right	−3.95 (4.83)	−5.15 (4.34)
Other-related socially threatening, left	−3.29 (4.36)	−3.04 (3.13)
Other-related socially threatening, right	−3.88 (4.87)	−4.58 (4.33)
Other-related physically threatening, left	−2.82 (3.76)	−2.86 (3.16)
Other-related physically threatening, right	−3.31 (4.07)	-4.57 (4.54)
Other-related neutral, left	−3.12 (4.38)	−2.82 (3.29)
Other-related neutral, right	−3.54 (4.88)	−4.72 (4.48)
*EPN (250-450 ms)*		
Self-related socially threatening, left	0.84 (3.07)	1.96 (2.89)
Self-related socially threatening, right	1.50 (3.02)	2.29 (3.11)
Self-related physically threatening, left	0.97 (2.72)	2.31 (2.54)
Self-related physically threatening, right	1.59 (2.62)	2.60 (2.84)
Self-related neutral, left	0.67 (3.12)	1.95 (2.86)
Self-related neutral, right	1.25 (2.95)	2.31 (3.21)
Other-related socially threatening, left	1.04 (2.85)	2.33 (2.61)
Other-related socially threatening, right	1.90 (2.50)	2.90 (2.92)
Other-related physically threatening, left	1.26 (2.38)	2.33 (2.51)
Other-related physically threatening, right	2.14 (1.94)	2.76 (3.09)
Other-related neutral, left	1.27 (2.79)	2.60 (2.32)
Other-related neutral, right	2.05 (2.65)	3.03 (2.76)
*P3b (300-450 ms)*		
Self-related socially threatening	1.67 (1.83)	1.16 (1.81)
Self-related physically threatening	1.63 (1.73)	1.59 (2.11)
Self-related neutral	1.55 (1.71)	0.95 (1.88)
Other-related socially threatening	1.47 (1.79)	0.98 (1.61)
Other-related physically threatening	1.54 (1.73)	1.41 (1.72)
Other-related neutral	1.31 (1.77)	1.41 (1.96)
LPP (450-600 ms)*		
Self-related socially threatening	2.09 (1.03)	2.08 (1.63)
Self-related physically threatening	2.05 (.95)	1.93 (1.41)
Self-related neutral	2.03 (1.11)	1.56 (1.56)
Other-related socially threatening	2.05 (1.17)	1.84 (1.42)
Other-related physically threatening	1.81 (1.11)	1.89 (1.40)
Other-related neutral	1.75 (1.06)	1.97 (1.56)

*Both samples consisted of *n* = 20 individuals.

### P100 (90-120 ms)

**Fig. 1:**
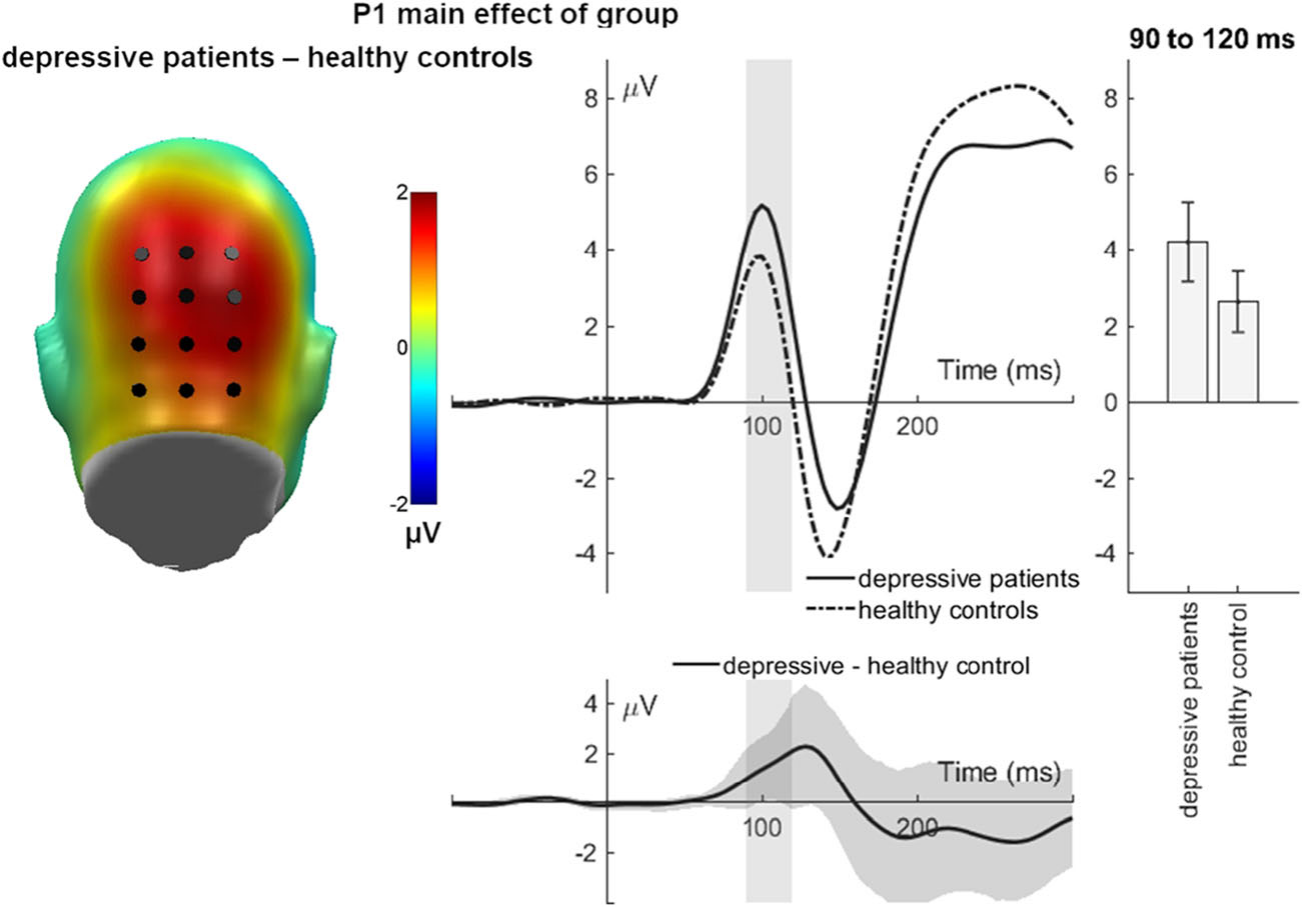
P1 main effect of group. The difference topographies display differences between depressive patients and controls, highlighting the P1 cluster. The electrode curve displays the mean activity over the occipital P1 cluster for both groups separately. The difference plot contains 95 % bootstrap confidence intervals of average group differences

For the P100, a significant main effect of group was found (*F*(1, 39) = 5.10, *p* = 0.030, partial η^2^ = 0.116). Depressive patients showed larger mean P100 amplitudes than healthy controls (Fig. 1). Further main or interaction effects were not significant (all *F*’s < 2.21; all *p*’s > 0.145; Table 4).

**Table 4:** *F*, *p*, and *η*^*2*^ values for the ANOVA analyzing mean P100 amplitudes (90-120 ms)

	*df*	*F*	*p*	*η*^*2*^
Self-reference	1, 39	2.21	.145	.054
Emotional valence	1.68, 65.33^a^	.65	.501	.016
Group	1, 39	5.10	.030*	.116
Self-reference x Emotional valence	2, 78	.47	.626	.012
Self-reference x Group	1, 39	.37	.550	.009
Emotional valence x Group	1.68, 65.33^a^	.21	.775	.005
Self-reference x Emotional valence x Group	2, 78	1.39	.254	.035

*p < 0.05, ^a^Greenhouse–Geisser corrected.

### N170 (120-170 ms)

**Fig. 2:**
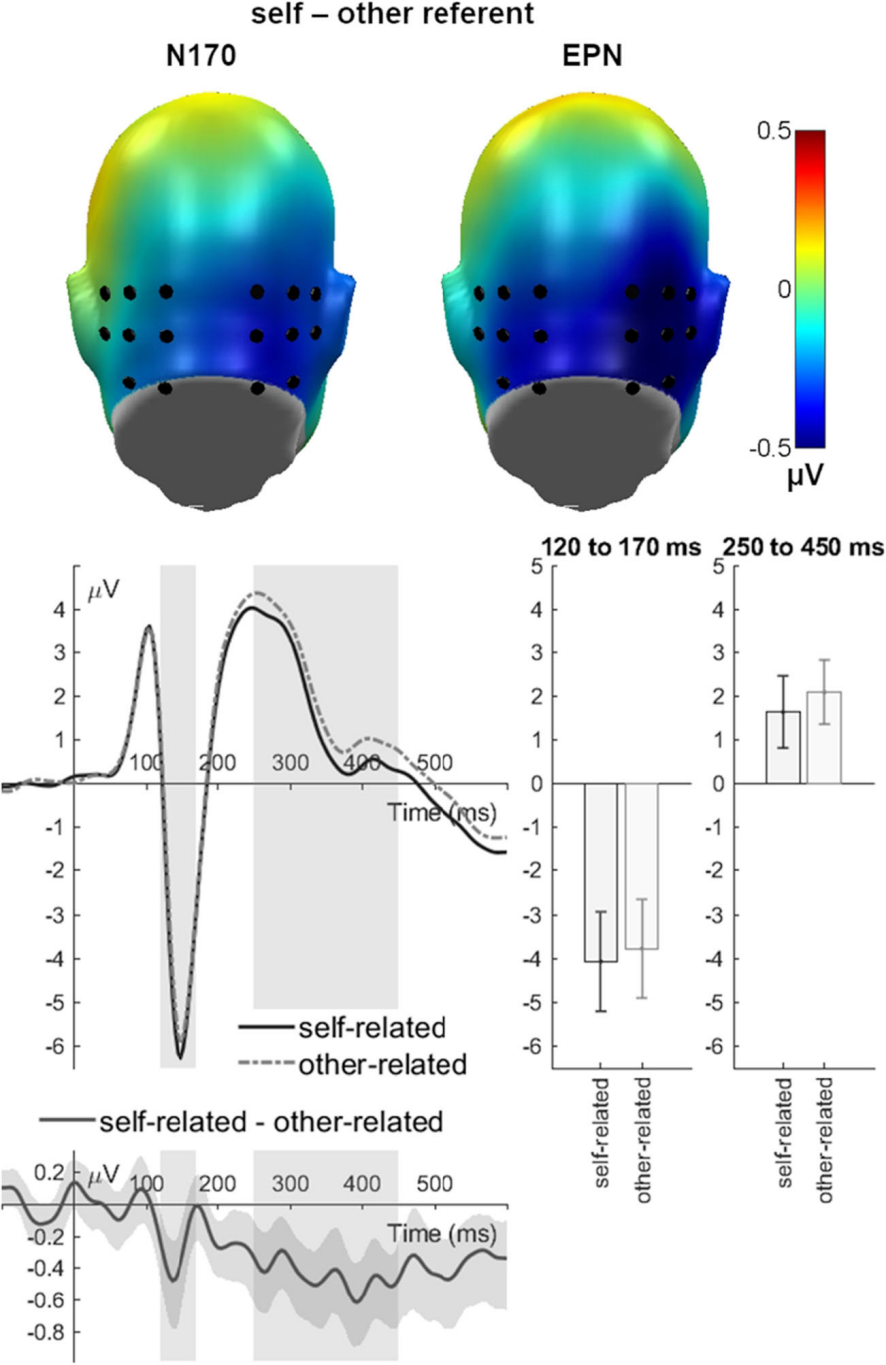
N170 and EPN main effects of reference. The difference topographies display differences between self- and other-related trials across patients and controls, highlighting the N170/EPN cluster. The electrode curve displays the mean activity over the occipital N170/EPN cluster. The difference plot contains 95 % bootstrap confidence intervals of intra-individual differences

Regarding the face-specific N170 component, the repeated measures ANOVA did not show any significant effect related to depression (all *F*’s < 2.36; all *p*’s > 0.101; Table 5). In all participants, however, a significant main effect of self-reference was found (*F*(1, 39) = 6.92, *p* = 0.012, partial η^2^ = 0.151; Fig. 2). Larger mean N170 amplitudes for faces in self-related compared to other-related conditions were found. Additionally, a significant main effect of laterality was found (*F*(1, 39) = 5.52, *p* = 0.024, partial η^2^ = 0.124). Here, mean N170 amplitudes were more negative over the right compared to the left electrode cluster. Further main or interaction effects did not reach significance (all *F*’s < 2.11; all *p*’s > 0.155).

**Table 5:** *F*, *p*, and *η*^*2*^ values for ANOVAs analyzing mean amplitudes of N170 and EPN components

	*df*	*F*	*p*	*η*^*2*^
*N170 (120-170 ms)*				
Self-reference	1, 39	6.92	0.012*	0.151
Emotional valence	2, 78	0.90	0.412	0.022
Laterality	1, 39	5.52	0.024*	0.124
Group	1, 39	0.14	0.709	0.004
Self-reference x Emotional valence	2, 78	0.97	0.385	0.024
Self-reference x Laterality	1, 39	2.11	0.155	0.051
Self-reference x Group	1, 39	<0.01	0.976	<0.001
Emotional valence x Laterality	2, 78	1.01	0.371	0.025
Emotional valence x Group	2, 78	0.31	0.732	0.008
Laterality x Group	1, 39	1.46	0.234	0.036
Self-reference x Emotional valence x Laterality	2, 78	0.41	0.668	0.010
Self-reference x Emotional valence x Group	2, 78	1.42	0.248	0.035
Self-reference x Laterality x Group	1, 39	<0.01	0.978	<0.001
Valence x Laterality x Group	2, 78	0.30	0.746	0.007
Self-reference x Emotional valence x Laterality x Group	2, 78	2.36	0.101	0.057
*EPN (250-450 ms)*				
Self-reference	1, 39	15.64	<0.001*	0.286
Emotional valence	2, 78	0.64	0.528	0.016
Laterality	1, 39	6.56	0.014*	0.144
Group	1, 39	1.83	0.184	0.045
Self-reference x Emotional valence	1.46, 57.07^a^	1.16	0.307	0.029
Self-reference x Laterality	1, 39	5.34	0.026*	0.120
Self-reference x Group	1, 39	0.05	0.822	0.001
Emotional valence x Laterality	2, 78	0.43	0.653	0.011
Emotional valence x Group	2, 78	0.17	0.841	0.016
Laterality x Group	1, 39	0.55	0.463	0.014
Self-reference x Emotional valence x Laterality	2, 78	0.09	0.912	0.002
Self-reference x Emotional valence x Group	1.46, 57.07^a^	0.38	0.619	0.010
Self-reference x Laterality x Group	1, 39	0.16	0.689	0.004
Emotional valence x Laterality x Group	2, 78	0.26	0.769	0.007
Self-reference x Emotional valence x Laterality x Group	2, 78	0.11	0.897	0.003

*p < 0.05, ^a^Greenhouse–Geisser corrected.

### EPN (250-450 ms)

For the EPN, depression did not show any significant main or interaction effects (all *F*’s < 1.83; all *p*’s > 0.184; Table 5), although a significant main effect of self-reference was found in the whole sample (*F*(1, 39) = 15.64, *p* < 0.001, partial η^2^ = 0.286; Fig. 2). Larger mean EPN amplitudes for faces in self-related conditions than in other-related conditions were found. In addition, the repeated measures ANOVA showed a significant main effect of laterality (*F*(1, 39) = 6.56, *p* = 0.014, partial η^2^ = 0.144). In contrast to the N170 time window, mean amplitudes were more negative over the left compared with the right electrode cluster. Furthermore, a significant interaction of self-reference and laterality was found (*F*(1, 39) = 5.34, *p* = 0.026, partial η^2^ = 0.120). The main effect of self-reference over the left electrode cluster (*F*(1, 39) = 7.28, *p* = 0.010, partial η^2^ = 0.157) was substantially smaller than over the right electrode cluster (*F*(1, 39) = 25.03, *p* < 0.001, partial η^2^ = 0.391). There were no further significant main or interaction effects (all *F*’s < 1.16; all *p*’s > 0.307).

### LPP (450-600 ms)

**Fig. 3:**
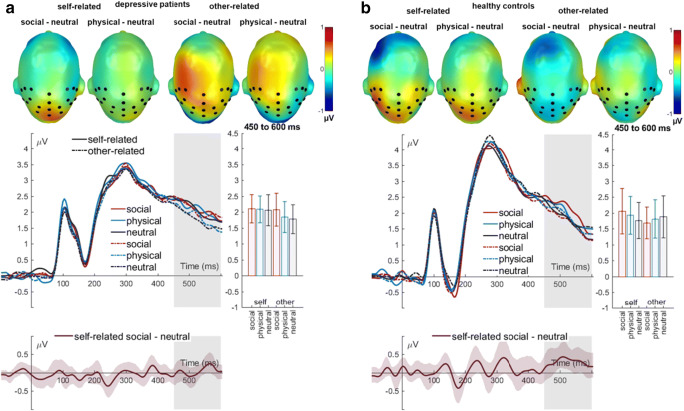
LPP interaction effect between group, reference, and emotional valence. The difference topographies displays differences between emotion categories for self- and other-related trials, highlighting the LPP cluster. **a**) Differences within depressive patients. Mean activity over the centro-parietal LPP cluster for all conditions. **b**) Differences within healthy controls. Mean activity over the centroparietal LPP cluster for all conditions. All difference plots contain 95% bootstrap confidence intervals of intra-individual differences

For the LPP over the centroparietal cluster, there was no significant main or two-way interaction effect related to depression (all *F*’s < 1.46; all *p*’s > 235; Table 6). Additionally, the ANOVA showed no significant main effect of emotional valence (*F*(2, 76) = 3.16, *p* = 0.059, partial η^2^ = 0.077). A significant three-way interaction of group, self-reference, and emotional valence was found (*F*(2, 76) = 4.30, *p* = 0.017, partial η^2^ = 0.102) (Fig. 3). Resolving this interaction, additional post-hoc 2 (self-reference) x 3 (emotional valence) repeated measures ANOVAs were conducted for groups separately. In the depressive group, the post-hoc ANOVA revealed a significant main effect of self-reference (*F*(1, 19) = 5.07, *p* = 0.036, partial η^2^ = 0.211). Depressive patients showed larger LPP responses to faces in self-related than in other related context. Furthermore, a significant main effect of emotional valence was found (*F*(2, 38) = 4.87, *p* = 0.013, partial η^2^ = 0.204). Post-hoc analyses showed that LPP responses to faces after socially threatening sentences were larger than to faces after neutral sentences (*t*(19) = 3.56, *p* = 0.006). There were no significant differences in the reactions to faces in socially threatening versus physically threatening contexts or physically threatening versus neutral contexts (all *t*’s < 1.99, all *p*s > 0.093). The interaction of self-reference and emotional valence did not reach significance (*F*(2, 38) = 1.36, *p* = 0.268, partial η^2^ = 0.067). Explorative FDR corrected post-hoc analyses revealed that depressive patients showed a tendency toward larger LPP responses to faces after other-related socially threatening sentences than after other-related neutral sentences (*t*(19) = 2.72, *p* = 0.070). Moreover, depressive patients showed more pronounced mean LPP amplitudes in reaction to faces in a self-related socially threatening and neutral context compared with the faces presented in an other-related neutral context (all *t*’s > 3.00; all *p*’s < 0.050). In addition, a tendency toward larger mean LPP amplitudes was found in reaction to faces after self-related physically threatening faces than in reaction to faces after other-related neutral sentences (*t*(19) = 2.39, *p* = 0.096). Additional significant *t*-tests were not found in depressive patients (all *t*’s < 2.31, all *p*s > 0.100). In healthy controls, the post-hoc repeated measures ANOVA showed no significant main effects of self-reference (*F*(2, 38) = 0.07, *p* = 0.799, partial η^2^ = 0.003) and emotional valence (*F*(2, 38) = 1.07, *p* = 0.338, partial η^2^ = 0.053, ε = 0.739). However, a significant interaction of self-reference and emotional valence was found (*F*(2, 38) = 3.27, *p* = 0.049, partial η^2^ = 0.147). FDR corrected post-hoc *t*-tests did not show any significant differences (all *t*’s < 2.28, all *p*’s > 0.510). However, without FDR correction, separate post-hoc analyses for self-reference conditions showed larger LPP responses for faces in self-related socially threatening contexts than for faces in self-related neutral contexts (*t*(19) = 2.28, *p* = 0.034), whereas no differences were detected in other-related contexts (all *t*’s < 0.80, all *p*’s > 0.433). Moreover, no significant main or interaction effects were found in the initial repeated measures ANOVA (all *F*’s < 1.17; all *p*’s > 0.317).

**Table 6:** *F*, *p*, and *η*^2^values for ANOVAs analyzing mean amplitudes of the LPP component

	*df*	*F*	*p*	*η*^*2*^
*LPP (450-600 ms)*				
Self-reference	1, 38	0.55	0.463	0.014
Emotional valence	1.64, 62.14^a^	3.16	0.059	0.077
Group	1, 38	0.05	0.825	0.001
Self-reference x Emotional valence	2, 76	1.17	0.317	0.030
Self-reference x Group	1, 38	1.46	0.235	0.037
Emotional valence x Group	1.64, 62.14^a^	0.30	0.741	0.008
Self-reference x Emotional valence x Group	2, 76	4.30	0.017*	0.102

*p < 0.05, ^a^Greenhouse–Geisser corrected.

### Exploratory analyses on childhood maltreatment and neural activity in patients with depression

In depressive patients, exploratory analyses showed significant correlations between the severity of maltreatment and the mean amplitudes at P100 and P3b stages. While there were significant positive associations of the CTQ sumscore, physical abuse, emotional abuse, and relational peer victimization with the mean amplitude of the P100 component, a significant negative correlation between physical abuse and the mean P3b amplitude was found (Table 7).

**Table 7:** Partial Spearman rank correlations between childhood maltreatment and mean electrocortical activity at P100 and P3b stages

	Depressive patients (*n* = 21)
	*Rho (p)*
P100	
CTQ sumscore	0.50 (0.025)*
Physical abuse	0.60 (0.005)*
Emotional abuse	0.55 (0.011)*
Physical neglect	0.42 (0.066)
Emotional neglect	0.36 (0.121)
Sexual abuse	0.22 (0.363)
Relational peer victimization	0.45 (0.046)*
P3b	
CTQ sumscore	−0.02 (0.925)
Physical abuse	−0.47 (0.038)*
Emotional abuse	−0.05 (0.833)
Physical neglect	−0.11 (0.648)
Emotional neglect	0.03 (0.911)
Sexual abuse	0.21 (0.381)
Relational peer victimization	−0.02 (0.921)

Partial correlations were calculated with Spearman rank correlations, severity of depressive symptoms (BDI) served as control variable; *p < 0.05.

## Discussion

In a study of contextual face processing, we found evidence for altered information processing in individuals with depression. By randomly pairing sentences and faces in each trial of the experiment, the present study was designed to investigate one-time and short-term affective context modulation effects (instead of contextual learning processes), which have been demonstrated before for healthy individuals (Klein et al., 2015). Across conditions, depressive patients showed an augmented mean P100 amplitude when confronted with neutral faces that were preceded by emotional context information. At the same time, mean LPP amplitudes of depressive patients were larger in response to faces presented in a self-related context than to faces presented in an other-related context. In addition, LPP amplitudes of depressive patients were more pronounced in response to faces presented after socially threatening sentences than in response to faces presented after neutral sentences. Furthermore, exploratory post-hoc analyses revealed that LPP amplitudes of depressive patients were modulated by emotional valence only when presented in an other-related context, while healthy controls rather showed differentiated mean LPP amplitudes in reaction to emotionally valenced faces when these were presented in a self-related context. Moreover, self-related conditions led to larger mean amplitudes than other-related conditions in both samples. However, contrary to our expectations, we could not find specific differences between depressive patients and healthy controls with regard to the manipulations of self-reference or emotional valence on N170 and EPN stages. Furthermore, exploratory analyses showed that among depressive patients a history of childhood maltreatment was associated with mean amplitudes at P100 and P3b stages.

### P100

In line with previous research (Auerbach et al., 2015; Yang et al., 2011), the P100 component was modulated by depression. However, against our hypotheses, P100 amplitudes were not specifically enhanced in reaction to self-related negative stimuli, but depressed patients presented with a generally heightened early responsiveness, leading to higher mean P100 amplitudes regardless of valence and self-relevance. Hence, instead of a generally attenuated responsiveness to affective stimuli (Foti et al., 2010) or a specifically amplified responsiveness for negative, self-referential stimuli (Auerbach et al., 2015; Scher et al., 2005), findings suggest a generalized early potentiation of encoding interpersonal information in depressive individuals. A possible explanation for the observed heightened responsiveness on the P100 may arise from findings of an impaired recognition ability of facial expressions in depressive patients in that they attributed emotional valence to affectively neutral faces (Leppänen et al., 2004). Similarly, meta-analyses on facial emotion expression processing in major depression revealed that individuals with depression are prone to evaluate positive, neutral, or ambiguous facial expressions more negatively than healthy controls and have impaired emotion recognition for every facial expression except sadness (Bourke et al., 2010; Dalili et al., 2015). With respect to the present study, especially when being confronted with negative and neutral context information, depressed patients could have suffered from a sustained negatively biased interpretation of the inherently neutral facial expressions presented in the experiment. This could have led to the observed intensified processing on the ERP level, according to the proposed negativity bias. This also would be in line with previous reports of depressive subjects responding faster to negative face expressions and selective attention toward threatening stimuli (Delle-Vigne et al., 2014; Mathews et al., 1996). Although this is a very first trial to examine the P100 component in the processing of facial stimuli in depressed subjects and replication is warranted, findings suggest that depressive individuals engage in early, automated processing of affective and referential information, which may be negatively biased and may serve to reinforce and intensify depressive symptoms.

### N170 and EPN

Contrasting with prior research and our hypotheses (Chen et al., 2014; Connell et al., 2015), depressive and healthy individuals did not show divergent N170 and EPN amplitudes dependent on differential context information. The functional meaning of the N170 and EPN as well as the paradigm that was used in the present study may play a crucial role in explaining missing effects on these components. As stated earlier, the P100 is suggested to reflect early categorization processes at a stage of face detection (Herrmann et al., 2005; Itier & Taylor, 2004; Liu et al., 2002), whereas the N170 component reflects configural and facial emotion-specific processing (Feuerriegel et al., 2014). The EPN has been connected with more enhanced emotional processing (Hajcak et al., 2010). Hence, for the modulation of N170 and EPN differences on emotional valence are required. With respect to the present paradigm, it may be suggested that the combination of different emotionally valenced sentences with neutral faces was not strong enough to evoke emotion-specific modulations of the N170 and EPN. That is, the faces may still have been received as emotionally neutral. Unfortunately, no affective ratings were conducted during the course of the current study; therefore, we cannot preclude nor confirm this hypothesis. In line with this assumption, however, prior studies pointed out that alterations of the N170 component in depressive patients were dependent on affective expressions of the faces (Foti et al., 2010; Jaworska et al., 2012). Hence, the neutral faces used in the present study, although combined with emotionally valenced sentences, do not seem to be sufficient to evoke differences on N170 and EPN stages between depressive patients and healthy controls. The process of face detection, reflected by the P100, however, may be more sensitive in depressive patients.

### LPP

Contrasting with previous studies (Foti et al., 2010; Kayser et al., 2000; MacNamara et al., 2016), depressive individuals did not show weaker LPP amplitudes than healthy controls, although differentiated processing of faces presented after emotionally valenced sentences in healthy controls and depressive patients could be found. In line with previous research (Wagner et al., 2015), depressive patients showed most pronounced LPP responses to faces with self-related context information and to faces with socially threatening information. However, against our hypotheses and previous literature (Auerbach et al., 2015; Burkhouse et al., 2017; Jaworska et al., 2012; Shestyuk & Deldin, 2010), LPP responses did not differ between emotional valence categories within the self-related condition, whereas depressive patients showed a tendency toward increased reactions to socially threatening stimuli when presented in an other-related context. Hence, it may be suggested that LPP reactions may be indicative of a rather generalized enhanced sensitivity in individuals with MDD. Exploratory analyses showed that mean LPP amplitudes in the self-related conditions were more pronounced than in the other-related neutral condition. On the premise that the other-related neutral condition may serve as a baseline condition, results may be considered in terms of a sustained negatively biased interpretation of neutral facial expressions, irrespective of the actual affective valence (Bourke et al., 2010; Dalili et al., 2015; Delle-Vigne et al., 2014; Mathews et al., 1996; Siegle et al., 2002). However, because LPP modulation is more sensitive to differences in self-reference (Klein et al., 2015; Wieser et al., 2014), the sustained negatively biased processing may not apply to other-related threats in general but may be restricted to socially threatening contexts. This, again, is in line with studies reporting a negativity bias in depressive individuals (Delle-Vigne et al., 2014; Mathews et al., 1996; Scher et al., 2005) addressing prior assumptions that processing of faces in depressive patients is particularly sensitive to social context information (Gilboa-Schechtman et al., 2002). Consequently, it may be assumed that depressive patients are more responsive to any negative social information, whether addressed to themselves or to other people. This proneness to process negative incoming social information may be crucial in the etiology and maintenance of depressive symptoms as it has been proposed in cognitive models of depression (Abramson et al., 1978; Beck, 1976; Bower, 1981; Teasdale & Dent, 1987).

In line with previous reports (Klein et al., 2015; Wieser et al., 2014), an interaction of self-reference and emotional valence could be detected in healthy individuals. Here, results were indicative of a stronger LPP modulation in self-relevant contexts with more pronounced mean amplitudes following neutral faces in a socially threatening context in self-related conditions. However, LPP findings in healthy individuals should be interpreted with caution because post-hoc comparisons did not withstand FDR correction for multiple comparisons.

### Replication of previous findings in healthy individuals

Because the present study sought to extend previous findings regarding the effect of context information on facial processing (Klein et al., 2015), it is important to note that previous results could be replicated to a high degree in the healthy control sample as well as in the overall sample of the present study. In both samples, self-relevant context cues led to most pronounced activations on the N170 and EPN components, a result that fits well with the results of past studies (Klein et al., 2015; Wieser et al., 2014). Similarly, in line with findings regarding the centroparietal LPP indicated by Klein et al. (2015), the interaction between self-reference and valence showed a significant effect in the healthy control sample. Contrasting with our hypotheses, neutral faces that have been associated with socially threatening information were not preferentially processed compared with physically threatening contexts in the total sample. At this point, using affective second-hand information may have lacked power to build up an observable unique pattern of activation. However, despite the question of a possible distinction between socially and physically threatening negative context information, the results indicate that context information is capable of modulating the processing of neutral target stimuli in depressive patients, most likely as a top-down regulation function of a higher order alerting system.

### Maltreatment related depression and its impact on emotion processing

On the basis of explorative analyses, the present study was one of the first to show significant associations of various kinds of childhood maltreatment including relational peer victimization with modulations of the P100 and P3b components in depressive patients. In line with previous studies (Dong et al., 2004; Gibb et al., 2007; Humphreys & Zeanah, 2015; McLaughlin et al., 2014), different kinds of maltreatment were differentially correlated with ERP modulations. With respect to the examined ERP components, findings of enhanced mean P100 amplitudes in maltreated depressive patients were in line with a previous study in physically abused children (Pollak & Tolley-Schell, 2003). This may be indicative of an enhanced selective attention for potentially threatening cues in maltreated depressive patients. Notably, findings indicate that in addition to physical and emotional abuse relational peer victimization may also cause alterations in responsivity to interpersonal stimuli. However, the negative association of mean P3b amplitudes with severity of physical abuse in depressive participants contrasts with previous findings of increased P3b in physically abused children (Pollak et al., 2001; Shackman et al., 2007). The age of the sample, the kind of paradigm, and current psychopathology may account for differences in the outcome of experiences of maltreatment on mean P3b amplitudes. In line with this assumption, Matz et al. (2010) could show that, regardless of the diagnosis, patients with adverse childhood experiences provided an attenuated cortical reaction to affective stimuli. Finally, the explorative analyses of the present study emphasize the importance of examining the differential impact of adverse life experiences on the development of depressive symptoms. Our findings support the idea of a maltreatment-related clinical subtype of depression (Andersen & Teicher, 2008; Zisook et al., 2007). As indicated in prior research (Pollak et al., 2001; Pollak & Tolley-Schell, 2003; Shackman et al., 2007), the P100 and P3b components seem promising candidates in marking this subtype on ERP level.

### Limitations

The present study and its findings are limited by the small sample size. Recruitment of increased sample sizes would allow for more reliable analyses. However, replication of previous findings as well as dependability and split-half reliability scores support the reliability of the presented results. Additionally, identification of the EPN component was impaired, because difference waves for the EPN did not show a profound negativity. In line with previous studies (Bruchmann et al., 2020; Schindler et al., 2019, 2020), the EPN was captured around the N2 wave amplitude. Therefore, comparability to previous studies using the same study design may be limited (Klein et al., 2015). However, explorative analyses using the EPN window applied in the prior study did not show differential effects. With respect to reliability scores presented in Table 2, the current identification is suggested to better capture the EPN in the present study. Additionally, due to unbalanced blink artifacts, i.e., many depressive patients consistently blinked after stimulus offset at approximately 800 ms post-stimulus, later LPP effects occurring after 600 ms post-stimulus could not be examined in the present study. To deal with the problem of multiple (implicit) comparisons inherent in ERP research, time windows and electrode clusters for ERP components were based on a priori hypotheses and validated by using a collapsed localizer (Luck & Gaspelin, 2017). While post-hoc analyses within ERP components were FDR corrected, however, we did not correct for multiple comparisons across ERP components in the present study. Hence, findings and conclusions are limited as we cannot preclude that they are based on false-positive results. Moreover, the restricted range of experiences of childhood maltreatment may impair the validity of the correlation analyses. Although correlation analyses were conducted for explorative purposes only. A further limitation is the lack of obtained affective ratings of the supposedly neutral faces during the course of the experiment. By including affective ratings at different time points of the experiment, future studies should be able to determine a possible change in the subjective affective interpretation of the neutral facial expressions and thereby reveal potentially biased information processing. Moreover, the current study lacks an evaluation of the different sentences through valence and arousal ratings. Although the set of sentences was evaluated in a prior study (Klein et al., 2015), it remains open whether this evaluation may be generalized to the current sample. Hence, all conclusions regarding valence effects have to be drawn with caution. Another limitation was that analyses were not controlled for comorbidities of the depressed patients, especially anxiety disorders. There are many studies that point out possible information processing biases, e.g., for PTSD (Javanbakht et al., 2011) or social anxiety disorder (Mueller et al., 2009). However, because there were no systematic comorbidities in our study, the impact of a specific disorder is not assessable in the present study. Nevertheless, exploratory correlation analyses examining associations between ERP components and subscales of the BSI in the depressive sample revealed potential differences in depressive patients in emotion processing with regards to the presence of symptoms of anxiety and psychoticism. Symptoms of anxiety and psychoticism were associated with diminished amplitudes on N170 and EPN components, which may indicate weakened emotional processing in a subgroup of patients with depression. With respect to this exploratory findings and because it is known that the majority of depressive patients suffer from comorbid disorders (Löwe et al., 2008), future studies should try to control for this aspect. It would be of particular interest to examine populations with specific combinations of comorbidities. To our knowledge, there is still a lack of research regarding the possible subtypes of depression and the respective information processing abnormalities. Because it has been shown that first episode MDD and recurrent MDD patients show differential facial processing (Chen et al., 2014), recurrence and number of depressive episodes may play an additional role in differential processing in subtypes of depression. In the present sample, however, recurrent and first episode MDD patients were merged. Because of the rather small sample size, we could not control systematically and adequately for potential effects of symptom recurrence. Although exploratory analyses did not reveal any effects of recurrence on ERPs in the present sample, this limitation has to be kept in mind when considering the present results. Furthermore, inter-rater reliability of structured clinical interviews administered by trained clinical psychologists was not assessed in terms of rescoring the information given in the interviews. However, administration of interviews was supervised by experienced senior therapists, which suggests reliable and valid diagnoses.

To sum up, our study is in line with previous research reporting altered emotional processing in depression. Interestingly, our study showed a distinct processing pattern, particularly with a generalized heightened initial responsiveness to interpersonal information. Further research is needed to clarify whether this is a result of facilitated attention processes, which may play a crucial role in the etiology of depressive disorder. Further studies also should disclose the association of cortical reactivity and childhood maltreatment in depressive patients.

## Supplementary Information

ESM 1 (DOCX 149 kb)

## References

[CR1] Abramson, L. Y., Seligman, M. E., & Teasdale, J. D. (1978). Learned helplessness in humans: critique and reformulation. *Journal of Abnormal Psychology*, 87(1), 49–74. 10.1037/0021-843X.87.1.49 649856

[CR2] American Psychiatric Association. (2000). *Diagnostic and statistical manual of mental disorders: DSM-IV-TR*. American Psychiatric Publishing, Inc.

[CR3] Andersen, S. L. & Teicher, M. H. (2008). Stress, sensitive periods and maturational events in adolescent depression*. Trends in Neurosciences*, 31(4), 183-191. 10.1016/j.tins.2008.01.004 18329735

[CR4] Auerbach, R. P., Stanton, C. H., Proudfit, G. H., & Pizzagalli, D. A. (2015). Self-referential processing in depressed adolescents: A high-density ERP study. *Journal of Abnormal Psychology*, 124(2), 233–245. 10.1037/abn0000023. 10.1037/abn0000023 PMC4429006 25643205

[CR5] Beck, A. T. (1976). Cognitive therapy and the emotional disorders.

[CR6] Beck, A. T., Rush, A. J., Shaw, B. F., & Emery, G. (1979). *Cognitive therapy of depression.* Guilford Press.

[CR7] Benjamini, Y., & Hochberg, Y. (1995). Controlling the false discovery rate: a practical and powerful approach to multiple testing. *Journal of the Royal Statistical Society Series B*, 57, 289–300. 10.2307/2346101.

[CR8] Bourke, C., Douglas, K., & Porter, R. (2010). Processing of facial emotion expression in major depression: a review. *Australian and New Zealand Journal of Psychiatry*, 44(8), 681-696. 10.3109/00048674.2010.496359 10.3109/00048674.2010.496359 20636189

[CR9] Bower, G. H. (1981). Mood and memory. *The American Psychologist*, 36(2), 129–148. 10.1037/0003-066X.36.2.129 10.1037//0003-066x.36.2.129 7224324

[CR10] Burkhouse, K. L., Owens, M., Feurer, C., Sosoo, E., Kudinova, A., & Gibb, B. E. (2017). Increased neural and pupillary reactivity to emotional faces in adolescents with current and remitted major depressive disorder. *Social Cognitive and Affective Neuroscience*, 12(5), 783–792. 10.1093/scan/nsw184 PMC5460039 28008074

[CR11] Bruchmann, M., Schindler, S., & Straube, T. (2020). The spatial frequency spectrum of fearful faces modulates early and mid-latency ERPs but not the N170. *Psychophysiology*, 57(9), e13597. 10.1111/psyp.13597 10.1111/psyp.13597 32390215

[CR12] Carpenter, B., Gelman, A., Hoffman, M. D., Lee, D., Goodrich, B., Betancourt, M., Brubaker, M., Guo, J., Li, P., & Riddell, A. (2017). Stan: A Probabilistic Programming Language. *Journal of Statistical Software*, 76(1), 1-32. 10.18637/jss.v076.i01 10.18637/jss.v076.i01 PMC9788645 36568334

[CR13] Chen, J., Ma, W., Zhang, Y., Wu, X., Wei, D., Liu, G., Deng, Z., Yang, L., & Zhang, Z. (2014). Distinct facial processing related negative cognitive bias in first-episode and recurrent major depression: evidence from the N170 ERP component. *PloS One*, 9(10), e109176. 10.1371/journal.pone.0109176 10.1371/journal.pone.0109176 PMC4196777 25314024

[CR14] Clayson, P. E., & Miller, G. A. (2017a). Psychometric considerations in the measurement of event-related brain potentials: Guidelines for measurement and reporting. *International Journal of Psychophysiology*, 111, 57-67. 10.1016/j.ijpsycho.2016.09.005 10.1016/j.ijpsycho.2016.09.005 27619493

[CR15] Clayson, P. E., & Miller, G. A. (2017b). ERP Reliability Analysis (ERA) Toolbox: An open-source toolbox for analyzing the reliability of event-related brain potentials. *International Journal of Psychophysiology*, 111, 68-79. 10.1016/j.ijpsycho.2016.10.012 10.1016/j.ijpsycho.2016.10.012 27769878

[CR16] Cohen, J. (1988). *Statistical power analysis for the behavioral sciences*. *Statistical Power Analysis for the Behavioral Sciences* (Vol. 2nd). 10.1234/12345678

[CR17] Connell, A. M., Patton, E., & McKillop, H. (2015). Binge Drinking, Depression, and Electrocortical Responses to Emotional Images. *Psychology of Addictive Behaviors*, 29(3), 673–682. 10.1037/adb0000071 10.1037/adb0000071 25915691

[CR18] Dai, Q., & Feng, Z. (2012). More excited for negative facial expressions in depression: evidence from an event-related potential study. *Clinical Neurophysiology : Official Journal of the International Federation of Clinical Neurophysiology*, 123(11), 2172–9. 10.1016/j.clinph.2012.04.018 10.1016/j.clinph.2012.04.018 22727714

[CR19] Dalili, M. N., Penton-Voak, I. S., Harmer, C. J., & Munafò, M. R. (2015). Meta-analysis of emotion recognition deficits in major depressive disorder. *Psychological Medicine*, 45(6), 1135–44. 10.1017/S0033291714002591 10.1017/S0033291714002591 PMC4712476 25395075

[CR20] Delle-Vigne, D., Wang, W., Kornreich, C., Verbanck, P., & Campanella, S. (2014). Emotional facial expression processing in depression: Data from behavioral and event-related potential studies. *Neurophysiologie Clinique*, 44, 169–187. 10.1016/j.neucli.2014.03.003 10.1016/j.neucli.2014.03.003 24930940

[CR21] Diéguez-Risco, T., Aguado, L., Albert, J., & Hinojosa, J. A. (2013). Faces in context: modulation of expression processing by situational information. *Social Neuroscience*, 8(6), 601–20. 10.1080/17470919.2013.834842 10.1080/17470919.2013.834842 24053118

[CR22] Dong, M., Anda, R. F., Felitti, V. J., et al. (2004). The interrelatedness of multiple forms of childhood abuse, neglect, and household dysfunction. *Child Abuse & Neglect*, 28, 771–84. 10.1016/j.chiabu.2004.01.008 10.1016/j.chiabu.2004.01.008 15261471

[CR23] Feuerriegel, D., Churches, O., Hofmann, J., & Keage, H. A. D. (2014). The N170 and face perception in psychiatric and neurological disorders: A systematic review. *Clinical Neurophysiology*. 10.1016/j.clinph.2014.09.015 10.1016/j.clinph.2014.09.015 25306210

[CR24] Fields, E. C., & Kuperberg, G. R. (2012). It’s All About You: An ERP study of emotion and self-relevance in discourse. *NeuroImage*, 62(1), 562–574. 10.1016/j.neuroimage.2012.05.003 10.1016/j.neuroimage.2012.05.003 PMC3678961 22584232

[CR25] Foti, D., Olvet, D. M., Klein, D. N., & Hajcak, G. (2010). Reduced electrocortical response to threatening faces in major depressive disorder. *Depression and Anxiety*, 27(March), 813–820. 10.1002/da.20712 10.1002/da.20712 20577985

[CR26] Franke, G. (2000). *BSI - Brief Symptom-Inventory - Deutsche Version. Manual.* Beltz.

[CR27] Frenkel, T. I., & Bar-Haim, Y. (2011). Neural activation during the processing of ambiguous fearful facial expressions: an ERP study in anxious and nonanxious individuals. *Biological Psychology*, 88(2–3), 188–95. 10.1016/j.biopsycho.2011.08.001 10.1016/j.biopsycho.2011.08.001 21846487

[CR28] Gibb, B. E., Chelminski, I., & Zimmerman, M. (2007). Childhood emotional, physical, and sexual abuse, and diagnoses of depressive and anxiety disorders in adult psychiatric outpatients. *Depression and Anxiety*, 24(4), 256–63. 10.1002/da.20238 10.1002/da.20238 17041933

[CR29] Gilboa-Schechtman, E., Erhard-Weiss, D., & Jeczemien, P. (2002). Interpersonal deficits meet cognitive biases: Memory for facial expressions in depressed and anxious men and women. *Psychiatry Research*, 113(3), 279–293. 10.1016/S0165-1781(02)00266-4 10.1016/s0165-1781(02)00266-4 12559484

[CR30] Gotlib, I. H., Krasnoperova, E., Yue, D. N., & Joormann, J. (2004). Attentional Biases for Negative Interpersonal Stimuli in Clinical Depression. *Journal of Abnormal Psychology*, 113(1), 127–135. 10.1037/0021-843X.113.1.121 10.1037/0021-843X.113.1.121 14992665

[CR31] Hajcak, G., Dunning, J. P., & Foti, D. (2009). Motivated and controlled attention to emotion: time-course of the late positive potential. *Clinical Neurophysiology : Official Journal of the International Federation of Clinical Neurophysiology*, 120(3), 505–10. 10.1016/j.clinph.2008.11.028 10.1016/j.clinph.2008.11.028 19157974

[CR32] Hajcak, G., MacNamara, A., & Olvet, D. M. (2010). Event-related potentials, emotion, and emotion regulation: an integrative review. *Developmental Neuropsychology*, 35(2), 129–55. 10.1080/87565640903526504 10.1080/87565640903526504 20390599

[CR33] Hautzinger, M., Keller, F., & Kühner, C. (2006). *Das Beck Depressionsinventar II. Deutsche Bearbeitung und Handbuch zum BDI II*. Harcourt Test Services.

[CR34] Herrmann, M. J., Ehlis, A.-C., Ellgring, H., & Fallgatter, A. J. (2005). Early stages (P100) of face perception in humans as measured with event-related potentials (ERPs). *Journal of Neural Transmission*, 112, 1073–1081. 10.1007/s00702-004-0250-8 10.1007/s00702-004-0250-8 15583954

[CR35] Hinojosa, J. A., Mercado, F., & Carretié, L. (2015). N170 sensitivity to facial expression: A meta-analysis. *Neuroscience and Biobehavioral Reviews*, 55, 498–509. 10.1016/j.neubiorev.2015.06.002 10.1016/j.neubiorev.2015.06.002 26067902

[CR36] Hirsh, J. B., & Inzlicht, M. (2008). The devil you know: Neuroticism predicts neural response to uncertainty. *Psychological Science*, 19(10), 962–967. 10.1111/j.1467-9280.2008.02183.x 10.1111/j.1467-9280.2008.02183.x 19000202

[CR37] Humphreys, K. L., & Zeanah, C. H. (2015). Deviations from the expectable environment in early childhood and emerging psychopathology. *Neuropsychopharmacolog*y, 40(1), 154–170. 10.1038/npp.2014.165. 10.1038/npp.2014.165 PMC4262894 24998622

[CR38] Iffland, B. & Neuner, F. (2020). Varying cognitive scars – differential associations between types of childhood maltreatment and facial emotion processing. *Frontiers in Psychology,* 11, 732. 10.3389/fpsyg.2020.00732 10.3389/fpsyg.2020.00732 PMC7177008 32373037

[CR39] Itier, R. J., & Taylor, M. J. (2004). N170 or N1? Spatiotemporal differences between object and face processing using ERPs. *Cerebral Cortex*, 14(2), 132–142. 10.1093/cercor/bhg111 10.1093/cercor/bhg111 14704210

[CR40] Javanbakht, A., Liberzon, I., Amirsadri, A., Gjini, K., & Boutros, N. N. (2011). Event-related potential studies of post-traumatic stress disorder: a critical review and synthesis. *Biology of Mood & Anxiety Disorders*, 1(1), 5. 10.1186/2045-5380-1-5 10.1186/2045-5380-1-5 PMC3377169 22738160

[CR41] Jaworska, N., Blier, P., Fusee, W., & Knott, V. (2012). The temporal electrocortical profile of emotive facial processing in depressed males and females and healthy controls. *Journal of Affective Disorders*, 136(3), 1072–81. 10.1016/j.jad.2011.10.047 10.1016/j.jad.2011.10.047 PMC3288478 22127390

[CR42] Kayser, J., Bruder, G. E., Tenke, C. E., Stewart, J. E., & Quitkin, F. M. (2000). Event-related potentials (ERPs) to hemifield presentations of emotional stimuli: Differences between depressed patients and healthy adults in P3 amplitude and asymmetry. *International Journal of Psychophysiology*, 36(3), 211–236. 10.1016/s0167-8760(00)00078-7 10754195

[CR43] Klein, F., Iffland, B., Schindler, S., Wabnitz, P., & Neuner, F. (2015). This person is saying bad things about you: The influence of physically and socially threatening context information on the processing of inherently neutral faces. *Cognitive, Affective, & Behavioral Neuroscience*, 15(4), 736–748. 10.3758/s13415-015-0361-8 10.3758/s13415-015-0361-8 25967930

[CR44] Klinitzke, G., Romppel, M., Häuser, W., Brähler, E., & Glaesmer, H. (2012). The German Version of the Childhood Trauma Questionnaire (CTQ): psychometric characteristics in a representative sample of the general population. *Psychotherapie, Psychosomatik, Medizinische Psychologie*, 62(2), 47–51. 10.1055/s-0031-1295495 10.1055/s-0031-1295495 22203470

[CR45] Kühner, C., Bürger, C., Keller, F., & Hautzinger, M. (2007). Reliabilität und validität des revidierten Beck- Depressionsinventars (BDI-II). Befunde aus deutschsprachigen stichproben. *Nervenarzt*, 78(6), 651–656. 10.1007/s00115-006-2098-7 10.1007/s00115-006-2098-7 16832698

[CR46] Langner, O., Dotsch, R., Bijlstra, G., Wigboldus, D. H. J., Hawk, S. T., & Van Knippenberg, A. (2010). Presentation and validation of the Radboud Faces Database. *Cognition & Emotion*, 24(8), 1377–1388. 10.1080/02699930903485076

[CR47] Leppänen, J. M. (2006). Emotional information processing in mood disorders: a review of behavioral and neuroimaging findings. *Current Opinion in Psychiatry*, 19(1), 34–39. 10.1097/01.yco.0000191500.46411.00 10.1097/01.yco.0000191500.46411.00 16612176

[CR48] Leppänen, J. M., Milders, M., Bell, J. S., Terriere, E., & Hietanen, J. K. (2004). Depression biases the recognition of emotionally neutral faces. *Psychiatry Research*, 128(2), 123–33. 10.1016/j.psychres.2004.05.020 10.1016/j.psychres.2004.05.020 15488955

[CR49] Litvak, V., Mattout, J., Kiebel, S. J., Phillips, C., Henson, R., Kilner, J. M., … Friston, K. J. (2011). EEG and MEG data analysis in SPM8. *Computational Intelligence and Neuroscience*, 2011, 852961. 10.1155/2011/852961 10.1155/2011/852961 PMC3061292 21437221

[CR50] Liu, J., Harris, A., & Kanwisher, N. (2002). Stages of processing in face perception: an MEG study. *Nature Neuroscience*, 5, 910–916. 10.1038/nn909 10.1038/nn909 12195430

[CR51] Löwe, B., Spitzer, R. L., Williams, J. B. W., Mussell, M., Schellberg, D., & Kroenke, K. (2008). Depression, anxiety and somatization in primary care: syndrome overlap and functional impairment. *General Hospital Psychiatry*, 30(3), 191–199. 10.1016/j.genhosppsych.2008.01.001 10.1016/j.genhosppsych.2008.01.001 18433651

[CR52] Luck, S. J., & Gaspelin, N. (2017). How to get statistically significant effects in any ERP experiment (and why you shouldn’t). *Psychophysiology*, 54(1), 146–157. 10.1111/psyp.12639 10.1111/psyp.12639 PMC5178877 28000253

[CR53] MacNamara, A., Kotov, R., & Hajcak, G. (2016). Diagnostic and symptom-based predictors of emotional processing in generalized anxiety disorder and major depressive disorder: An event-related potential study. *Cognitive Therapy and Research*, 40(3), 275–289. 10.1007/s10608-015-9717-1. 10.1007/s10608-015-9717-1 PMC4916772 27346901

[CR54] Mathews, A., Ridgeway, V., & Williamson, D. A. (1996). Evidence for attention to threatening stimuli in depression. *Behaviour Research and Therapy*, 34(9), 695–705. 10.1016/0005-7967(96)00046-0 10.1016/0005-7967(96)00046-0 8936752

[CR55] Matz, K., Junghöfer, M., Elbert, T., Weber, K., Wienbruch, C., & Rockstroh, B. (2010). Adverse experiences in childhood influence brain responses to emotional stimuli in adult psychiatric patients. *International Journal of Psychophysiology*, 75(3), 277–286. 10.1016/j.ijpsycho.2009.12.010 10.1016/j.ijpsycho.2009.12.010 20045438

[CR56] McLaughlin, K. A., Sheridan, M. A., & Lambert, H. K. (2014). Childhood adversity and neural development: Deprivation and threat as distinct dimensions of early experience. *Neuroscience and Biobehavioral Reviews*, 47(11), 578–591. 10.1016/j.neubiorev.2014.10.012. 10.1016/j.neubiorev.2014.10.012 PMC4308474 25454359

[CR57] Mueller, E. M., Hofmann, S. G., Santesso, D. L., Meuret, A. E., Bitran, S., & Pizzagalli, D. A. (2009). Electrophysiological evidence of attentional biases in social anxiety disorder. *Psychological Medicine,* 39(7), 1141-1152. 10.1017/S0033291708004820 10.1017/S0033291708004820 PMC3204217 19079826

[CR58] Peyk, P., De Cesarei, A., & Junghöfer, M. (2011). Electro Magneto Encephalograhy Software: overview and integration with other EEG/MEG toolboxes. *Computational Intelligence and Neuroscience*, 201. 10.1155/2011/861705 PMC3090751 21577273

[CR59] Pollak, S. D., Klorman, R., Thatcher, J. E., & Cicchetti, D. (2001). P3b reflects maltreated children's reactions to facial displays of emotion. *Psychophysiology,* 38(2), 267-274. 11347872

[CR60] Pollak, S. D., & Tolley-Schell, S. A. (2003). Selective attention to facial emotion in physically abused children. *Journal of Abnormal Psychology,* 112(3), 323-338. 10.1037/0021-843x.112.3.323 12943012

[CR61] Righart, R., & de Gelder, B. (2006). Context influences early perceptual analysis of faces--an electrophysiological study. *Cerebral Cortex (New York, N.Y. : 1991)*, 16(9), 1249–57. 10.1093/cercor/bhj066 10.1093/cercor/bhj066 16306325

[CR62] Righart, R., & de Gelder, B. (2008). Rapid influence of emotional scenes on encoding of facial expressions: An ERP study. *Social Cognitive and Affective Neuroscience*, 3(3), 270–278. 10.1093/scan/nsn021 10.1093/scan/nsn021 PMC2566764 19015119

[CR63] Roth, M. C., Humphreys, K. L, King, L. S 1 ., & Gotlib, I. H. (2018). Self-Reported Neglect, Amygdala Volume, and Symptoms of Anxiety in Adolescent Boys. *Child Abuse & Neglect*, 80, 80-89. 10.1016/j.chiabu.2018.03.016. 10.1016/j.chiabu.2018.03.016 PMC5953811 29574295

[CR64] Sansen, L. M., Iffland, B., Catani, C., & Neuner, F. (2013). Entwicklung und evaluation des fragebogens zu belastenden sozialerfahrungen in der peergroup (FBS) [Development and evaluation of a questionnaire on stressful social experiences in peer groups (FBS)]. *Zeitschrift Fur Klinische Psychologie Und Psychotherapie*, 42(1), 34–44. 10.1026/1616-3443/a000184

[CR65] Schindler, S., Bruchmann, M., Bublatzky, F., & Straube, T. (2019). Modulation of face- and emotion-selective ERPs by the three most common types of face image manipulations. *Social Cognitive and Affective Neuroscience*, 14(5), 493–503. 10.1093/scan/nsz027 10.1093/scan/nsz027 PMC6545565 30972417

[CR66] Schindler, S., Bruchmann, M., Steinweg, A.-L., Moeck, R., & Straube, T. (2020). Attentional conditions differentially affect early, intermediate and late neural responses to fearful and neutral faces. *Social Cognitive and Affective Neuroscience*, 15(7), 765–774. 10.1093/scan/nsaa098 10.1093/scan/nsaa098 PMC7511883 32701163

[CR67] Scher, C. D., Ingram, R. E., & Segal, Z. V. (2005). Cognitive reactivity and vulnerability: empirical evaluation of construct activation and cognitive diatheses in unipolar depression. *Clinical Psychology Review*, 25(4), 487–510. 10.1016/j.cpr.2005.01.005 10.1016/j.cpr.2005.01.005 15914266

[CR68] Schupp, H. T., Flaisch, T., Stockburger, J., & Junghöfer, M. (2006). Chapter 2 Emotion and attention: event-related brain potential studies. *Progress in Brain Research*. 10.1016/S0079-6123(06)56002-9 10.1016/S0079-6123(06)56002-9 17015073

[CR69] Schupp, H. T., Öhman, A., Junghöfer, M., Weike, A. I., Stockburger, J., & Hamm, A. O. (2004). The facilitated processing of threatening faces: an ERP analysis. *Emotion*, 4(2), 189. 10.1037/1528-3542.4.2.189 15222855

[CR70] Shackman, J. E., Shackman, A. J., & Pollak, S. D. (2007). Physical abuse amplifies attention to threat and increases anxiety in children. *Emotion, 7*(4), 838-852. 10.1037/1528-3542.7.4.838 18039053

[CR71] Sharpley, C. F., & Bitsika, V. (2014). Validity, reliability and prevalence of four “clinical content” subtypes of depression. *Behavioural Brain Research*, 259, 9–15. 10.1016/j.bbr.2013.10.032 10.1016/j.bbr.2013.10.032 24172219

[CR72] Sheehan DV, Lecrubier Y, Sheehan KH, Amorim P, Janavs J, Weiller E, Hergueta T, Baker R, Dunbar GC. (1998). The Mini-International Neuropsychiatric Interview (M.I.N.I.): the development and validation of a structured diagnostic psychiatric interview for DSM-IV and ICD-10. *Journal of Clinical Psychiatry*, 59, 22-33. 9881538

[CR73] Sheline, Y. I., Barch, D. M., Price, J. L., Rundle, M. M., Vaishnavi, S. N., Snyder, A. Z., … Raichle, M. E. (2009). The default mode network and self-referential processes in depression. *Proceedings of the National Academy of Sciences of the United States of America*, 106(6), 1942–7. 10.1073/pnas.0812686106 10.1073/pnas.0812686106 PMC2631078 19171889

[CR74] Shestyuk, A. Y., & Deldin, P. J. (2010). Automatic and strategic representation of the self in major depression: Trait and state abnormalities. *The American Journal of Psychiatry*, 167, 536-544. 10.1176/appi.ajp.2009.06091444 10.1176/appi.ajp.2009.06091444 20360316

[CR75] Shestyuk, A.Y., Deldin, P.J., Brand, J.E., & Deveney, C.M. (2005). Reduced sustained brain activity during processing of positive emotional stimuli in major depression. *Biological Psychiatry*, 57(10), 1089–1096. 10.1016/j.biopsych.2005.02.013 10.1016/j.biopsych.2005.02.013 15866547

[CR76] Siegle, G. J., Steinhauer, S. R., Thase, M. E., Stenger, V. A., & Carter, C. S. (2002). Can’t shake that feeling: event-related fMRI assessment of sustained amygdala activity in response to emotional information in depressed individuals. *Biological Psychiatry*, 51(9), 693–707. 10.1016/s0006-3223(02)01314-8 11983183

[CR77] Stan Development Team. (2016). CmdStan: The command-line interface to Stan, version 2.10.0. http://mc-stan.org/

[CR78] Suslow T, Dannlowski U, Lalee-Mentzel J, Donges, U.-S., Arolt, V., & Kersting, A. (2004). Spatial processing of facial emotion in patients with unipolar depression: a longitudinal study. *Journal of Affective Disorders*, 83, 59–63. 10.1016/j.jad.2004.03.003 10.1016/j.jad.2004.03.003 15546646

[CR79] Teasdale, J. D., & Dent, J. (1987). Cognitive vulnerability to depression: an investigation of two hypotheses. *The British Journal of Clinical Psychology / the British Psychological Society*, 26 *(Pt 2)*, 113–126. 10.1111/j.2044-8260.1987.tb00737.x 10.1111/j.2044-8260.1987.tb00737.x 3580646

[CR80] Teicher, M. H., & Samson, J. A. (2013). Childhood maltreatment and psychopathology: A case for ecophenotypic variants as clinically and neurobiologically distinct subtypes. *American Journal of Psychiatry*, 170(10), 1114–1133. 10.1176/appi.ajp.2013.12070957 10.1176/appi.ajp.2013.12070957 PMC3928064 23982148

[CR81] Wagner, G., Schachtzabel, C., Peikert, G., & Bär, K.-J. (2015). The neural basis of the abnormal self-referential processing and its impact on cognitive control in depressed patients. *Human Brain Mapping*, 36(7), 2781–2794. 10.1002/hbm.22807 10.1002/hbm.22807 PMC6869596 25872899

[CR82] Wessing, I., Rehbein, M. A., Postert, C., Fürniss, T., & Junghöfer, M. (2013). The neural basis of cognitive change: reappraisal of emotional faces modulates neural source activity in a frontoparietal attention network. *NeuroImage*, 81, 15–25. 10.1016/j.neuroimage.2013.04.117 10.1016/j.neuroimage.2013.04.117 23664945

[CR83] Wieser, M. J., & Brosch, T. (2012). Faces in context: a review and systematization of contextual influences on affective face processing. *Frontiers in Psychology*, 3, 471. 10.3389/fpsyg.2012.00471 10.3389/fpsyg.2012.00471 PMC3487423 23130011

[CR84] Wieser, M. J., Gerdes, A. B. M., Büngel, I., Schwarz, K. A., Mühlberger, A., & Pauli, P. (2014). Not so harmless anymore: How context impacts the perception and electrocortical processing of neutral faces. *NeuroImage*, 92, 74-82. 10.1016/j.neuroimage.2014.01.022 10.1016/j.neuroimage.2014.01.022 24462933

[CR85] Wieser, M. J., Pauli, P., Reicherts, P., & Mühlberger, A. (2010). Don’t look at me in anger! Enhanced processing of angry faces in anticipation of public speaking. *Psychophysiology*, 47(2), 271–80. 10.1111/j.1469-8986.2009.00938.x 10.1111/j.1469-8986.2009.00938.x 20030758

[CR86] Wingenfeld, K., Spitzer, C., Mensebach, C., Grabe, H., Hill, A., Gast, U., Schlosser, N., Höpp, H., Beblo, T., & Driessen, M. (2010). Die deutsche Version des Childhood Trauma Questionnaire (CTQ): Erste Befunde zu den psychometrischen Kennwerten. *PPmP Psychotherapie Psychosomatik Medizinische Psychologie*, 60(11), 442–450. 10.1055/s-0030-1247564 10.1055/s-0030-1247564 20200804

[CR87] Wittchen, H. U., Zaudig, M., & Fydrich, T. (1997). *Strukturiertes Klinisches Interview für DSM-IV. Achse I und II*. Hogrefe.

[CR88] Yang, W., Zhu, X., Wang, X., Wu, D., & Yao, S. (2011). Time course of affective processing bias in major depression: an ERP study. *Neuroscience Letters*, 487(3), 372–377. 10.1016/j.neulet.2010.10.059 21036200

[CR89] Zeanah, C. H., & Sonuga-Barke, E. J. S. (2016). Editorial: The effects of early trauma and deprivation on human development - from measuring cumulative risk to characterizing specific mechanisms. Journal of Child Psychology and Psychiatry, 57(10), 1099–1102. 10.1111/jcpp.12642. 10.1111/jcpp.12642 27647049

[CR90] Zisook, S., Rush, A. J., Lesser, I., Wisniewski, S. R., Trivedi, M., Husain, M. M. et al. (2007). Preadult onset vs. adult onset of major depressive disorder: a replication study*. Acta Psychiatrica Scandinavica*, 115(3), 196-205. 10.1111/j.1600-0447.2006.00868.x 17302619

