# Supplementary material for: “She finds you abhorrent” - The impact of emotional context information on the cortical processing of neutral faces in depression
Source: Cogn Affect Behav Neurosci. 2021 Mar 15;21(2):426–44. doi: 10.3758/s13415-021-00877-x (PMC8121719; doi:10.3758/s13415-021-00877-x)
Supplement: Supplementary file 1 — (DOCX 149 kb) [file 13415_2021_877_MOESM1_ESM.docx]

| Supplement 1: Bivariate Spearman rank correlations between measures of childhood maltreatment and psychopathology and ERP mean amplitudes (*N* =41). | | | | | | | | | | | | | |
| --- | --- | --- | --- | --- | --- | --- | --- | --- | --- | --- | --- | --- | --- |
|  |  |  |  |  |  |  |  |  |  |  |  |  |  |
| P100 | mean | Self-related socially threatening | Self-related neutral | Self-related physically threatening | Other-related socially threatening | Other-related neutral | Other-related physically threatening |  |  |  |  |  |  |
|  | *Rho* | *Rho* | *Rho* | *Rho* | *Rho* | *Rho* | *Rho* |  |  |  |  |  |  |
| Childhood trauma questionnaire | .413^**^ | .420^**^ | .393^*^ | .402^**^ | .291 | .444^**^ | .330^*^ |  |  |  |  |  |  |
| Emotional abuse | .326^*^ | .311^*^ | .332^*^ | .309^*^ | .252 | .410^**^ | .265 |  |  |  |  |  |  |
| Sexual abuse | .135 | .113 | .129 | .133 | .059 | .221 | .029 |  |  |  |  |  |  |
| Physical abuse | .357^*^ | .312^*^ | .323^*^ | .293 | .355^*^ | .422^**^ | .290 |  |  |  |  |  |  |
| Emotional neglect | .409^**^ | .447^**^ | .418^**^ | .404^**^ | .294 | .366^*^ | .360^*^ |  |  |  |  |  |  |
| Physical neglect | .336^*^ | .351^*^ | .287 | .337^*^ | .197 | .396^*^ | .250 |  |  |  |  |  |  |
| Relational peer victimization | .475^**^ | .464^**^ | .527^**^ | .428^**^ | .375^*^ | .487^**^ | .493^**^ |  |  |  |  |  |  |
| Beck Depression Inventory | .453^**^ | .454^**^ | .486^**^ | .428^**^ | .383^*^ | .369^*^ | .498^**^ |  |  |  |  |  |  |
| Brief Symptom Inventory | .371^*^ | .379^*^ | .398^**^ | .359^*^ | .277 | .317^*^ | .395^*^ |  |  |  |  |  |  |
| BSI somatization | .374^*^ | .362^*^ | .376^*^ | .391^*^ | .379^*^ | .311^*^ | .444^**^ |  |  |  |  |  |  |
| BSI obsessive-compulsive | .282 | .312^*^ | .308^*^ | .287 | .191 | .232 | .287 |  |  |  |  |  |  |
| BSI interpersonal Sensitivity | .267 | .274 | .293 | .233 | .179 | .238 | .291 |  |  |  |  |  |  |
| BSI depression | .359^*^ | .357^*^ | .384^*^ | .350^*^ | .259 | .279 | .348^*^ |  |  |  |  |  |  |
| BSI anxiety | .228 | .248 | .267 | .245 | .171 | .229 | .223 |  |  |  |  |  |  |
| BSI hostility | .196 | .229 | .210 | .196 | .142 | .136 | .230 |  |  |  |  |  |  |
| BSI phobia | .284 | .305 | .290 | .193 | .312^*^ | .219 | .295 |  |  |  |  |  |  |
| BSI paranoia | .201 | .182 | .197 | .202 | .140 | .178 | .218 |  |  |  |  |  |  |
| BSI psychoticism | .404^**^ | .444^**^ | .426^**^ | .392^*^ | .339^*^ | .355^*^ | .432^**^ |  |  |  |  |  |  |
|  |  |  |  |  |  |  |  |  |  |  |  |  |  |
| N170 | mean | Self-related socially threatening. left | Self-related socially threatening. right | Self-related neutral. left | Self-related neutral. right | Self-related physically threatening. left | Self-related physically threatening. right | Other-related socially threatening. left | Other-related socially threatening. right | Other-related neutral. left | Other-related neutral. right | Other-related physically threatening. left | Other-related physically threatening. right |
|  | *Rho* | *Rho* | *Rho* | *Rho* | *Rho* | *Rho* | *Rho* | *Rho* | *Rho* | *Rho* | *Rho* | *Rho* | *Rho* |
| Childhood trauma questionnaire | -.066 | -.083 | -.078 | -.025 | -.025 | -.155 | -.104 | -.042 | -.027 | -.060 | .003 | -.146 | -.082 |
| Emotional abuse | .001 | .034 | -.012 | .036 | .005 | -.119 | -.064 | .025 | .026 | .042 | .065 | -.024 | -.028 |
| Sexual abuse | .020 | -.109 | .028 | -.049 | .057 | -.116 | .032 | -.063 | .116 | -.064 | .090 | -.095 | .048 |
| Physical abuse | .074 | .075 | .026 | .141 | .059 | .126 | .057 | .165 | .055 | .139 | .098 | .100 | -.006 |
| Emotional neglect | -.035 | -.060 | -.010 | -.011 | .031 | -.158 | -.056 | -.064 | .012 | -.070 | .046 | -.144 | -.018 |
| Physical neglect | .010 | -.016 | -.035 | .052 | .038 | -.052 | -.067 | .060 | .011 | .013 | .034 | -.060 | -.028 |
| Relational peer victimization | .228 | .172 | .248 | .210 | .257 | .078 | .212 | .163 | .219 | .261 | .313^*^ | .206 | .260 |
| Beck Depression Inventory | .256 | .145 | .300 | .195 | .313^*^ | .127 | .245 | .162 | .256 | .187 | .313^*^ | .219 | .311^*^ |
| Brief Symptom Inventory | .203 | .082 | .226 | .176 | .262 | .079 | .178 | .112 | .215 | .104 | .247 | .143 | .229 |
| BSI somatization | .127 | .047 | .200 | .061 | .179 | .032 | .137 | -.007 | .155 | .038 | .165 | .073 | .174 |
| BSI obsessive-compulsive | .197 | .076 | .251 | .139 | .283 | .059 | .204 | .093 | .240 | .060 | .236 | .112 | .259 |
| BSI interpersonal Sensitivity | .129 | .007 | .143 | .089 | .174 | -.022 | .071 | .070 | .143 | .043 | .178 | .075 | .144 |
| BSI depression | .258 | .189 | .242 | .252 | .291 | .154 | .218 | .205 | .239 | .194 | .280 | .220 | .253 |
| BSI anxiety | .285 | .144 | .336^*^ | .203 | .366^*^ | .101 | .297 | .127 | .340^*^ | .094 | .329^*^ | .164 | .346^*^ |
| BSI hostility | .066 | -.070 | .124 | .057 | .152 | -.074 | .072 | -.010 | .130 | -.048 | .134 | -.019 | .106 |
| BSI phobia | .127 | .083 | .100 | .159 | .155 | .106 | .088 | .138 | .128 | .140 | .156 | .126 | .120 |
| BSI paranoia | .031 | -.057 | .027 | .028 | .061 | .000 | .026 | .012 | .052 | -.053 | .045 | .016 | .036 |
| BSI psychoticism | .280 | .184 | .265 | .268 | .315^*^ | .175 | .213 | .235 | .265 | .208 | .304 | .218 | .272 |
|  |  |  |  |  |  |  |  |  |  |  |  |  |  |
| EPN | mean | Self-related socially threatening. left | Self-related socially threatening. right | Self-related neutral. left | Self-related neutral. right | Self-related physically threatening. left | Self-related physically threatening. right | Other-related socially threatening. left | Other-related socially threatening. right | Other-related neutral. left | Other-related neutral. right | Other-related physically threatening. left | Other-related physically threatening. right |
|  | *Rho* | *Rho* | *Rho* | *Rho* | *Rho* | *Rho* | *Rho* | *Rho* | *Rho* | *Rho* | *Rho* | *Rho* | *Rho* |
| Childhood trauma questionnaire | .111 | .117 | .150 | .085 | .140 | -.053 | .061 | .054 | .133 | .116 | .189 | .021 | .097 |
| Emotional abuse | .178 | .146 | .184 | .152 | .213 | .055 | .160 | .115 | .218 | .231 | .237 | .094 | .140 |
| Sexual abuse | .043 | .035 | .063 | .062 | .094 | .006 | .099 | .007 | .048 | .072 | .143 | .025 | .082 |
| Physical abuse | -.005 | -.019 | -.038 | .054 | .043 | -.087 | .016 | .016 | -.002 | .043 | .112 | -.113 | -.058 |
| Emotional neglect | .120 | .105 | .184 | .085 | .183 | -.061 | .090 | .042 | .154 | .087 | .200 | .026 | .161 |
| Physical neglect | .182 | .204 | .191 | .152 | .125 | .036 | .071 | .148 | .147 | .178 | .177 | .151 | .108 |
| Relational peer victimization | .114 | .054 | .058 | .150 | .155 | .091 | .067 | .064 | .149 | .128 | .084 | .063 | .104 |
| Beck Depression Inventory | -.094 | -.134 | -.053 | -.130 | -.060 | -.245 | -.122 | -.149 | -.102 | -.124 | .003 | -.158 | -.010 |
| Brief Symptom Inventory | -.045 | -.103 | -.010 | -.088 | -.022 | -.255 | -.106 | -.088 | -.042 | -.137 | .012 | -.126 | .037 |
| BSI somatization | -.116 | -.106 | -.058 | -.192 | -.151 | -.205 | -.167 | -.153 | -.133 | -.107 | -.071 | -.151 | -.080 |
| BSI obsessive-compulsive | -.021 | -.008 | .026 | -.062 | -.041 | -.172 | -.096 | -.052 | -.046 | -.101 | -.010 | -.071 | .033 |
| BSI interpersonal Sensitivity | -.053 | -.128 | -.038 | -.030 | .010 | -.244 | -.107 | -.080 | -.022 | -.098 | .044 | -.131 | .001 |
| BSI depression | -.012 | -.009 | .002 | -.003 | .016 | -.170 | -.068 | -.003 | -.020 | -.050 | .009 | -.053 | .043 |
| BSI anxiety | .145 | .079 | .213 | .032 | .155 | -.076 | .134 | .043 | .149 | -.026 | .152 | .041 | .258 |
| BSI hostility | .015 | -.082 | .103 | -.112 | .019 | -.288 | -.040 | -.068 | .021 | -.106 | .088 | -.098 | .081 |
| BSI phobia | -.099 | -.109 | -.086 | -.104 | -.093 | -.200 | -.140 | -.134 | -.128 | -.133 | -.040 | -.190 | -.034 |
| BSI paranoia | -.149 | -.162 | -.128 | -.154 | -.128 | -.300 | -.202 | -.126 | -.126 | -.216 | -.076 | -.216 | -.086 |
| BSI psychoticism | .044 | .016 | .055 | .024 | .021 | -.086 | -.041 | -.003 | .014 | .008 | .054 | -.022 | .061 |
|  |  |  |  |  |  |  |  |  |  |  |  |  |  |
| P3b | mean | Self-related socially threatening | Self-related neutral | Self-related physically threatening | Other-related socially threatening | Other-related neutral | Other-related physically threatening |  |  |  |  |  |  |
|  | *Rho* | *Rho* | *Rho* | *Rho* | *Rho* | *Rho* | *Rho* |  |  |  |  |  |  |
| Childhood trauma questionnaire | .005 | .096 | .142 | .008 | -.048 | -.055 | .016 |  |  |  |  |  |  |
| Emotional abuse | -.066 | -.038 | .017 | -.009 | -.169 | -.060 | -.045 |  |  |  |  |  |  |
| Sexual abuse | -.144 | -.060 | .003 | -.181 | -.088 | -.209 | -.120 |  |  |  |  |  |  |
| Physical abuse | -.239 | -.154 | -.123 | -.245 | -.283 | -.277 | -.248 |  |  |  |  |  |  |
| Emotional neglect | .009 | .108 | .121 | -.001 | .012 | -.054 | .014 |  |  |  |  |  |  |
| Physical neglect | .041 | .116 | .126 | .103 | -.055 | -.018 | .037 |  |  |  |  |  |  |
| Relational peer victimization | -.028 | -.054 | .014 | -.046 | -.044 | .000 | -.002 |  |  |  |  |  |  |
| Beck Depression Inventory | -.068 | -.010 | -.019 | -.086 | -.034 | -.143 | -.118 |  |  |  |  |  |  |
| Brief Symptom Inventory | -.099 | -.006 | -.006 | -.135 | -.045 | -.179 | -.154 |  |  |  |  |  |  |
| BSI somatization | -.072 | -.091 | -.045 | -.091 | -.062 | -.096 | -.116 |  |  |  |  |  |  |
| BSI obsessive-compulsive | -.120 | -.040 | -.056 | -.166 | -.057 | -.163 | -.179 |  |  |  |  |  |  |
| BSI interpersonal Sensitivity | -.042 | .073 | .026 | -.064 | -.049 | -.113 | -.079 |  |  |  |  |  |  |
| BSI depression | -.166 | -.089 | -.062 | -.195 | -.115 | -.234 | -.201 |  |  |  |  |  |  |
| BSI anxiety | -.209 | -.142 | -.127 | -.210 | -.112 | -.295 | -.271 |  |  |  |  |  |  |
| BSI hostility | -.046 | .080 | -.012 | -.035 | .017 | -.106 | -.138 |  |  |  |  |  |  |
| BSI phobia | .094 | .104 | .181 | .038 | .159 | .003 | .061 |  |  |  |  |  |  |
| BSI paranoia | -.066 | .036 | .026 | -.138 | -.017 | -.135 | -.098 |  |  |  |  |  |  |
| BSI psychoticism | -.059 | -.017 | -.005 | -.076 | -.031 | -.133 | -.086 |  |  |  |  |  |  |
|  |  |  |  |  |  |  |  |  |  |  |  |  |  |
| LPP | mean | Self-related socially threatening | Self-related neutral | Self-related physically threatening | Other-related socially threatening | Other-related neutral | Other-related physically threatening |  |  |  |  |  |  |
|  | *Rho* | *Rho* | *Rho* | *Rho* | *Rho* | *Rho* | *Rho* |  |  |  |  |  |  |
| Childhood trauma questionnaire | .147 | .158 | .318^*^ | .239 | .088 | .111 | .061 |  |  |  |  |  |  |
| Emotional abuse | .193 | .206 | .293 | .205 | .168 | .202 | .157 |  |  |  |  |  |  |
| Sexual abuse | -.035 | -.020 | .039 | -.036 | -.032 | -.056 | -.048 |  |  |  |  |  |  |
| Physical abuse | .031 | .061 | .136 | .002 | -.098 | .043 | .016 |  |  |  |  |  |  |
| Emotional neglect | .102 | .135 | .287 | .227 | .105 | .044 | -.019 |  |  |  |  |  |  |
| Physical neglect | .113 | .091 | .250 | .255 | .017 | .071 | .041 |  |  |  |  |  |  |
| Relational peer victimization | -.006 | .023 | .101 | .000 | -.013 | -.031 | .021 |  |  |  |  |  |  |
| Beck Depression Inventory | -.075 | -.055 | .075 | -.027 | -.008 | -.166 | -.190 |  |  |  |  |  |  |
| Brief Symptom Inventory | -.040 | -.035 | .124 | .011 | .008 | -.137 | -.163 |  |  |  |  |  |  |
| BSI somatization | .001 | .058 | .069 | -.013 | .067 | -.023 | -.066 |  |  |  |  |  |  |
| BSI obsessive-compulsive | -.037 | -.052 | .058 | .040 | -.010 | -.109 | -.159 |  |  |  |  |  |  |
| BSI interpersonal Sensitivity | -.068 | -.059 | .088 | -.016 | -.062 | -.142 | -.151 |  |  |  |  |  |  |
| BSI depression | -.144 | -.173 | .016 | -.101 | -.079 | -.211 | -.241 |  |  |  |  |  |  |
| BSI anxiety | .016 | -.004 | .120 | .047 | .086 | -.085 | -.087 |  |  |  |  |  |  |
| BSI hostility | .037 | .105 | .150 | .123 | .067 | -.057 | -.139 |  |  |  |  |  |  |
| BSI phobia | .216 | .155 | .290 | .167 | .192 | .149 | .171 |  |  |  |  |  |  |
| BSI paranoia | -.037 | -.025 | .137 | -.015 | .009 | -.113 | -.138 |  |  |  |  |  |  |
| BSI psychoticism | .012 | -.033 | .140 | .060 | .060 | -.064 | -.056 |  |  |  |  |  |  |
| Note: * *p* < .05, *** p* < .05, **** p* < .001. | | | | | | | | | | | | | |

| Supplement 2: *F,* *p,* and *η2* values for ANOVAs analyzing mean amplitudes of N170, EPN, and LPP components in the healthy control group (*n* = 20). | | | | | |
| --- | --- | --- | --- | --- | --- |
|  |  |  |  |  |  |
|  |  | *df* | *F* | *p* | *η^2^* |
|  |  |  |  |  |  |
| *N170 (120-170ms)* |  |  |  |  |  |
| Self-reference |  | 1, 19 | 3.50 | .077 | .155 |
| Emotional valence |  | 2, 38 | .75 | .481 | .038 |
| Laterality |  | 1, 19 | 6.21 | .022* | .246 |
| Self-reference x Emotional valence |  | 2, 38 | .32 | .729 | .016 |
| Self-reference x Laterality |  | 1, 19 | 1.20 | .287 | .059 |
| Emotional valence x Laterality |  | 2, 38 | 1.64 | .207 | .080 |
| Self-reference x Emotional valence x Laterality |  | 2, 38 | 1.47 | .243 | .072 |
|  |  |  |  |  |  |
| *EPN (250-450ms)* |  |  |  |  |  |
| Self-reference |  | 1, 19 | 6.45 | .020* | .254 |
| Emotional valence |  | 2, 38 | .21 | .816 | .011 |
| Laterality |  | 1, 19 | 1.57 | .226 | .076 |
| Self-reference x Emotional valence |  | **1.30, 24.74^a^** | .57 | .500 | .029 |
| Self-reference x Laterality |  | 1, 19 | 1.92 | .182 | .092 |
| Emotional valence x Laterality |  | **1.43, 27.23^a^** | .27 | .695 | .014 |
| Self-reference x Emotional valence x Laterality |  | 2, 38 | .15 | .965 | .008 |
|  |  |  |  |  |  |
| *LPP (450-600ms)* |  |  |  |  |  |
| Self-reference |  | 1, 19 | .07 | .799 | .003 |
| Emotional valence |  | **1.48, 28.10^a^** | 1.07 | .338 | .053 |
| Self-reference x Emotional valence |  | 2, 38 | 3.27 | .049* | .147 |
| Note: * *p* < .05, **^a^Greenhouse–Geisser corrected.** | | | | | |
